# Comparative Metabolic Effects of Glibenclamide in Type 2 Diabetes Mellitus: A Systematic Review and Meta-Analysis of Randomized Controlled Trials

**DOI:** 10.3390/life16101754

**Published:** 2026-10-20

**Authors:** Juan José Valenzuela-Fuenzalida, Antonia Jaña-Barria, Ana Ferrer-Contreras, Carolina Bustos-Muñoz, Maria Fernanda Delgado-Retamal, Mario Paillao, Alvaro Becerra-Farfan, Mathias Orellana-Donoso, Alejandro Bruna-Mejias, Gloria Cifuentes-Suazo, Maria P. Moya, Maria Piagkou, Vitor E. Valenti, Juan Sanchis-Gimeno, Jose E. León-Rojas, Jessica Paola Loaiza-Giraldo

**Affiliations:** 1 Department of Morphology, Faculty of Medicine, Andrés Bello University, Santiago 8370146, Chile; 2 Escuela de Fonoaudiología & Departamento de Ciencias Química y Biológicas, Facultad de Ciencias de la Salud, Universidad Bernardo O’Higgins, Santiago 8370854, Chile; alvaro.becerra@ubo.cl; 3 Escuela de Medicina, Universidad Finis Terrae, Santiago 7501015, Chile; mathor94@gmail.com; 4 Faculty of Medicine and Science, Universidad San Sebastián, Santiago 8420524, Chile; 5 Facultad de Medicina, Carrera de Odontología, Universidad Católica de la Santísima Concepción, Av. Alonso de Ribera 2850, Concepción 4090541, Chile; 6 Facultad de Ciencias de la Salud, Universidad Autónoma de Chile, Santiago 8910060, Chile; 7 Department of Anatomy, School of Medicine, Faculty of Health Sciences, National and Kapodistrian University of Athens, 10679 Athens, Greece; 8 Systematic Reviews Center for Cardiovascular and Metabolic Health, School of Philosophy and Sciences, São Paulo State University, Marília 01599-999, SP, Brazil; 9 GIAVAL Research Group, Department of Anatomy and Human Embryology, Faculty of Medicine, University of Valencia, 46001 Valencia, Spain; 10 Cerebro, Emoción y Conducta (CEC) Research Group, Escuela de Medicina, Universidad de las Américas (UDLA), Quito 170124, Ecuador; 11 Facultad de Ciencias de la Salud, Universidad Central del Valle del Cauca (UCEVA), Tuluá 763022, Valle del Cauca, Colombia

**Keywords:** glibenclamide, sulfonylureas, type 2 diabetes mellitus, meta-analysis, randomized controlled trials, metabolic outcomes

## Abstract

**Background:** Glibenclamide remains widely used for glycemic control in type 2 diabetes mellitus (T2DM), but its comparative metabolic effects across glycemic, lipid, anthropometric, and cardiometabolic outcomes remain uncertain. We conducted a systematic review and meta-analysis of randomized controlled trials (RCTs) comparing glibenclamide with other antidiabetic therapies. **Methods:** We restricted eligibility to randomized trials in adults with T2DM. We performed random-effects meta-analyses for continuous outcomes; we assessed statistical heterogeneity using I^2^, risk of bias using Cochrane RoB 2, and certainty of evidence using GRADE. **Results:** We retained 19 reports representing 18 unique randomized trials for the qualitative synthesis; after final verification of study design and population, 14 reports, each from a distinct trial, contributed data to at least one quantitative meta-analysis. RoB 2 was assessed for a principal eligible result from all 19 included reports (18 unique trials): 11 report-level results (57.9%) were at high overall risk of bias, and eight (42.1%) raised some concerns. Within the 14-report quantitative subset, 10 were at high overall risk of bias, and four raised some concerns. No significant difference was observed for HbA1c (MD −0.45%; 95% CI −1.05 to 0.16; I^2^ = 99%) or fasting plasma glucose (MD −1.52 mmol/L; 95% CI −3.27 to 0.23; I^2^ = 99%). Body weight was higher with glibenclamide (MD 2.41 kg; 95% CI 1.63 to 3.20; I^2^ = 91%), and triglycerides showed a small nominal increase (MD 1.11 mg/dL; 95% CI 0.02 to 2.19; I^2^ = 91%); both findings were supported by very low-certainty evidence and were not uniformly stable in leave-one-out analyses. **Conclusions:** Across randomized T2DM trials, the available evidence does not demonstrate consistent differences in most metabolic outcomes between glibenclamide and heterogeneous comparator therapies. Interpretation is limited by substantial clinical and statistical heterogeneity, high risk of bias, imprecision, and predominantly low or very low certainty of evidence. The pooled estimates should not be interpreted as evidence of therapeutic equivalence between drug classes, and comparative safety remains uncertain because hypoglycemia was reported inconsistently.

## 1. Introduction

Type 2 diabetes mellitus (T2DM) is a chronic metabolic disorder with a strong inflammatory component, characterized by persistent hyperglycemia resulting from a variable combination of insulin resistance and progressive pancreatic β-cell dysfunction [1]. It is estimated that approximately 537 million adults worldwide are living with diabetes, of whom nearly 90% have T2DM. The burden is disproportionately higher in low- and middle-income countries, where access to newer glucose-lowering therapies may be limited [2]. Although traditionally considered a disease of middle-aged and older adults, the incidence of T2DM has increased among younger populations in parallel with obesity, physical inactivity, and energy-dense diets [3]. Interindividual variability in inflammatory signaling may also contribute to phenotypic heterogeneity in T2DM; human association studies involving NLRP3-related pathways illustrate this broader biological variability, although the trials synthesized here did not evaluate these genetic factors [3].

From a pathophysiological perspective, T2DM initially involves a compensatory increase in insulin secretion that maintains near-normal glucose levels despite peripheral insulin resistance. Over time, chronic exposure to hyperglycemia, lipotoxicity, and pro-inflammatory mediators leads to progressive β-cell dysfunction, loss of functional β-cell mass, and irreversible impairment of insulin secretion [3]. This process is mediated, in part, by oxidative stress and activation of pro-apoptotic signaling pathways induced by an adverse metabolic milieu [4]. At clinical diagnosis, it is estimated that 40% to 80% of β-cell function has already been lost; however, a residual fraction of β-cell mass may be partially recovered with adequate and timely glycemic control [2].

Among pharmacological strategies for treating T2DM, sulfonylureas have historically played a central role because of their potent glucose-lowering efficacy and low cost. Their primary mechanism of action involves inhibition of ATP-sensitive potassium (K_ATP) channels in pancreatic β-cells, leading to membrane depolarization, opening of voltage-dependent calcium channels, and stimulation of insulin secretion [2]. Despite their effectiveness, sulfonylureas are consistently associated with clinically relevant adverse effects, particularly hypoglycemia and weight gain, raising concerns regarding their long-term safety profile [2].

Glibenclamide (glyburide) is one of the most widely prescribed sulfonylureas worldwide and exerts its effect by binding to sulfonylurea receptor 1 (SUR1), closing K_ATP channels and stimulating insulin secretion that is partially independent of ambient glucose concentrations [5]. In addition to its pancreatic effects, glibenclamide has been shown to reduce hepatic glucose production, contributing further to glycemic control [1]. However, due to its high affinity for mitochondrial KATP channels, glibenclamide has been associated with an increased risk of prolonged hypoglycemia. Previous observational studies and comparative analyses have also suggested a higher incidence of major adverse cardiovascular events (MACE) than with other second-generation sulfonylureas, such as gliclazide and glimepiride; however, these findings rely on external evidence and were not evaluated in the randomized controlled trials included in the present review [2].

Despite these concerns, glibenclamide remains widely used in many regions, particularly in settings with constrained healthcare resources or limited access to newer antidiabetic agents. Nevertheless, evidence on its metabolic effects beyond glycemic control remains fragmented and heterogeneous, largely from individual randomized trials with limited sample sizes or studies focused on specific populations. Moreover, previous evidence syntheses have often focused on selected clinical settings, such as gestational diabetes, or evaluated isolated outcomes without integrating glycemic, lipid, anthropometric, inflammatory, and insulin resistance parameters or providing a structured appraisal of the overall certainty of evidence. Given the persistent clinical use of glibenclamide despite the availability of alternative therapies with potentially favorable metabolic and safety profiles, a comprehensive, updated evaluation of its effectiveness and safety is warranted. Therefore, this systematic review and meta-analysis aimed to assess the comparative metabolic efficacy of glibenclamide and summarize available safety data from randomized controlled trials in adults with type 2 diabetes mellitus, while incorporating a structured assessment of evidence certainty using the GRADE approach.

## 2. Methods

### 2.1. Protocol and Registration

This systematic review and meta-analysis was conducted and reported in accordance with the Preferred Reporting Items for Systematic Reviews and Meta-Analyses (PRISMA) guidelines [6]. We prospectively registered the study protocol in the International Prospective Register of Systematic Reviews (PROSPERO) under registration number CRD420261292101. During peer-review re-verification, we refined the final eligibility criteria to restrict the evidence set to randomized controlled trials enrolling adults with T2DM, excluding GDM populations and non-randomized studies. We introduced this eligibility refinement to ensure consistency between the review title and objective and the final evidence synthesized.

### 2.2. Literature Search

The search strategy combined keywords and controlled vocabulary terms related to the intervention and condition of interest, including “glibenclamide,” “glyburide,” “sulfonylurea,” “type 2 diabetes mellitus,” “T2DM,” and “clinical trial.” Boolean operators were applied as appropriate. In MEDLINE, the filters “Clinical Trial” and “Randomized Controlled Trial” were applied. For SCOPUS, Web of Science, and Google Scholar, we incorporated combinations of terms such as “randomized controlled trial,” “clinical trial,” “randomized,” and “trial” into the search strategy and verified them during screening. In CINAHL, we applied publication-type filters for clinical trials and randomized controlled trials when available. Two reviewers (JJ.V-F and JPL-G) independently screened the titles and abstracts of all retrieved records to identify potentially eligible studies. Full-text articles were obtained for studies deemed relevant by either reviewer. The reviewers resolved disagreements through discussion; when they could not reach consensus, a third reviewer (PN-B) adjudicated. The review team did not search EMBASE or CENTRAL because these databases were not accessible.

### 2.3. Study Selection

The final eligibility criteria were: adults diagnosed with type 2 diabetes mellitus (T2DM); intervention with glibenclamide (glyburide) at an approved dose or regimen; a randomized controlled design with an eligible comparator (placebo, standard care, or another antidiabetic therapy); and reporting of at least one relevant clinical, biochemical, or safety outcome. During the final re-review prompted by peer-review comments, we rechecked the original reports for population and allocation methods. We excluded studies enrolling gestational diabetes mellitus (GDM) or other forms of diabetes, as well as observational or non-randomized studies, from the final T2DM RCT evidence set. We retained randomized crossover trials in the qualitative synthesis when relevant but did not pool them when the reported data did not permit a valid paired effect estimate compatible with the prespecified meta-analytic approach. For reporting purposes, we distinguished publications/reports from unique randomized trials; multiple publications arising from the same randomized trial were counted as separate reports but as a single study/trial.

Studies were excluded if they were letters, case reports or case series, narrative or systematic reviews, non-human or in vitro studies; did not evaluate glibenclamide as part of the randomized treatment contrast; lacked an eligible randomized comparator; enrolled a non-T2DM population; or provided insufficient or incompatible outcome data for quantitative synthesis. Final design verification specifically excluded observational or non-randomized reports, and the GDM reports were removed from the primary T2DM synthesis. Reasons for potentially eligible full-text exclusions are summarized in Supplementary Table S3.

### 2.4. Data Extraction and Risk of Bias Assessment

Two reviewers (AL-FC and AF-JB) independently extracted data from eligible studies using a standardized form. Extracted variables included author and year, study design, sample size, participant characteristics, intervention and comparator, dose and duration, relevant outcomes, effect estimates, geographic region, and sex distribution. We reassessed risk of bias using the Cochrane Risk of Bias 2 (RoB 2) tool [7] for a principal eligible result from each of the 19 included reports, representing 18 unique randomized trials. These assessments therefore covered both the 14 reports contributing to quantitative synthesis and the five reports retained only for narrative synthesis. Because RoB 2 is result-specific, we assessed separate publications from the same underlying trial at the report/result level. We resolved disagreements through discussion and, when needed, third-reviewer adjudication (JP-LG).

RoB 2 evaluates five domains: bias arising from the randomization process (D1); bias due to deviations from intended interventions (D2); bias due to missing outcome data (D3); bias in outcome measurement (D4); and bias in selection of the reported result (D5). We judged each domain as low risk of bias, some concerns, or high risk of bias. We derived the overall judgment from the least favorable domain: low risk required low risk in all domains; some concerns indicated at least one domain with some concerns and no high-risk domain; and high risk indicated at least one high-risk domain or multiple domains with concerns that substantially lowered confidence in the result. Disagreements were resolved through discussion and, when consensus could not be achieved, a third reviewer (JP-LG) adjudicated.

### 2.5. Data Synthesis and Analysis of Scales

All statistical analyses were conducted in accordance with the Cochrane Handbook for Systematic Reviews of Interventions. For continuous outcomes retained in the final quantitative synthesis, effect sizes were expressed as mean differences (MDs) with corresponding 95% confidence intervals (CIs) when studies reported outcomes using the same measurement scale, including systolic and diastolic blood pressure, HbA1c (%), fasting plasma glucose (FPG), fructosamine, fasting insulin, HOMA-IR, body mass index (BMI), body weight, total cholesterol, LDL-C, HDL-C, triglycerides, and C-reactive protein (CRP). When outcomes were reported using different scales, calculation methods, or variability metrics across studies, we calculated effect sizes as standardized mean differences (SMDs) with 95% CIs; we applied this approach specifically to mean amplitude of glycemic excursions (MAGE). We considered postprandial plasma glucose and ApoB during extraction but did not retain them as pooled outcomes after final eligibility verification, as only one eligible randomized T2DM study remained for each endpoint.

The following outcomes were included in the final quantitative synthesis: systolic and diastolic blood pressure, HbA1c, fasting plasma glucose (FPG), fructosamine, fasting insulin, HOMA-IR, mean amplitude of glycemic excursions (MAGE), body mass index (BMI), body weight, total cholesterol, LDL-C, HDL-C, triglycerides, and C-reactive protein (CRP). We removed postprandial plasma glucose from quantitative synthesis because, excluding the GDM study, only one eligible T2DM RCT remained for that endpoint. We likewise did not pool ApoB after final design verification, as only one eligible randomized T2DM study remained. We generated forest plots for the 15 outcomes with at least two eligible RCTs (Figure 2 and Figure 3).

For continuous outcomes, we preferentially used final values for quantitative synthesis when available. When only change-from-baseline values were reported, we included these data according to Cochrane Handbook recommendations, provided pooling was clinically and statistically appropriate. When outcome measures were reported in different units, we converted values to a common unit before pooling when appropriate and clinically justified, using standard conversion factors based on established clinical laboratory conventions. When standard deviations were not directly reported, they were derived from available confidence intervals, standard errors, *p*-values, or other reported measures of dispersion using formulas recommended in the Cochrane Handbook for Systematic Reviews of Interventions. For fasting plasma glucose (FPG), we harmonized and pooled extracted values in mmol/L; we checked figure axes and reported pooled estimates against this common unit.

We did not include studies lacking sufficient information for effect size calculation in quantitative synthesis. We handled missing outcome data at the study level according to the information reported by the original investigators. We did not impute missing participant-level outcome data. We excluded trials with insufficient numerical data for extraction or transformation from the corresponding meta-analysis.

We assessed statistical heterogeneity using Cochran’s Q test and quantified it with the I^2^ statistic, interpreting values of <25%, 25–50%, and >50% as low, moderate, and substantial heterogeneity, respectively. Because the included comparator therapies represented different pharmacological classes and treatment contexts, we used a random-effects model (DerSimonian–Laird method) for all meta-analyses. We retained global pooled estimates to provide an overall descriptive summary of the randomized evidence, not to imply equivalence or interchangeability among comparator classes. Comparator-class subgroup analyses were not considered reliable because most classes were represented by too few trials; therefore, we interpret all global estimates as exploratory summaries, with clinical heterogeneity explicitly considered in the Results and Discussion. Where data permitted, we reported subgroup analyses by follow-up duration.

We performed leave-one-out sensitivity analyses by sequentially excluding individual studies. We planned to assess small-study effects when at least ten studies were available for a given meta-analysis. HbA1c (k = 13) and FPG (k = 10) met this numerical threshold in the final dataset; however, extreme statistical heterogeneity and substantial clinical diversity in comparator classes, treatment duration, and trial populations would make funnel-plot asymmetry and formal small-study-effect tests difficult to interpret. We therefore did not perform formal funnel-plot asymmetry or small-study-effect tests and retain the absence of a formal publication-bias assessment as a limitation. We did not retain meta-regression in the final analysis because, after restricting to eligible randomized T2DM trials, the study counts available for most outcome–moderator combinations were too small to support stable multivariable or repeated univariable moderator inference. A two-sided *p*-value < 0.05 was considered statistically significant.

### 2.6. Rating the Quality of Evidence

We assessed the certainty of evidence for each outcome using the Grading of Recommendations Assessment, Development and Evaluation (GRADE) approach. Certainty was classified as high, moderate, low, or very low after consideration of risk of bias, inconsistency, indirectness, imprecision, and publication bias. We used GRADEpro GDT to prepare the Summary of Findings table, presented in Supplementary Table S4 [8].

## 3. Results

### 3.1. Study Selection

**Figure 1:**
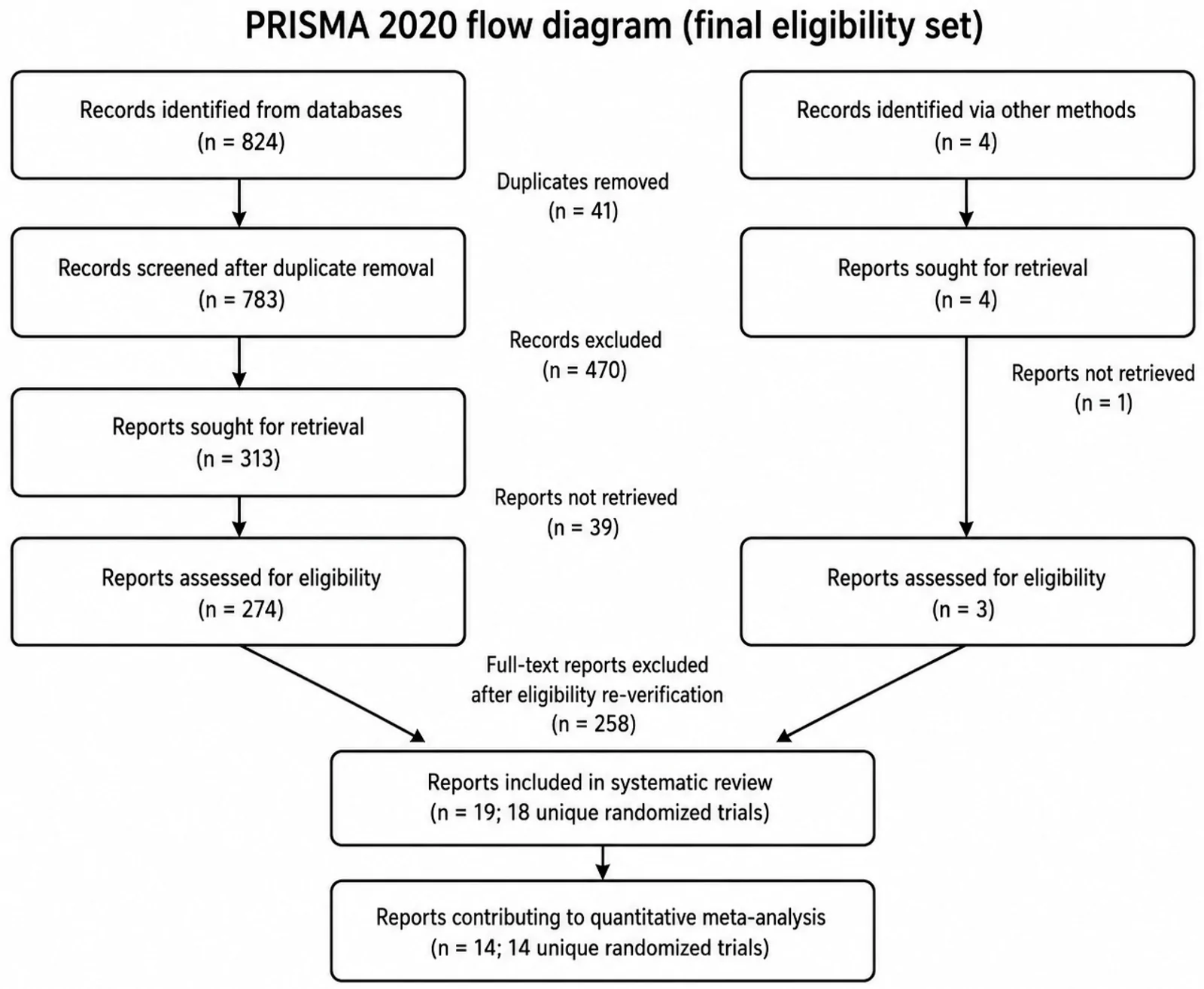
PRISMA 2020 flow diagram after final verification of study population and randomized design; the final set comprises 19 reports from 18 unique randomized trials, with 14 reports from 14 unique trials contributing to quantitative synthesis.

Figure 1 summarizes the study-selection process, and Supplementary Table S3 details the principal full-text exclusion reasons. The electronic database search identified 824 records, and we identified four additional records through other methods. After removing duplicates and screening, we assessed 277 reports for eligibility across the two identification pathways. Total screening articles is 32 [9,10,11,12,13,14,15,16,17,18,19,20,21,22,23,24,25,26,27,28,29,30,31,32,33,34,35,36,37,38,39,40]. Fourteen reports from 14 unique trials [9,11,12,13,15,16,17,19,21,22,23,24,25,26] provided sufficiently comparable and extractable data for at least one quantitative meta-analysis (Figure 4, Figure 5, Figure 6, Figure 7, Figure 8, Figure 9, Figure 10, Figure 11, Figure 12, Figure 13, Figure 14, Figure 15, Figure 16, Figure 17 and Figure 18). Five additional reports were retained for narrative synthesis; four represented additional unique trials, whereas Sposito et al. (2021) [39] and Wolf et al. (2021) [26] were separate reports from the same ADDENDA-BHS2 randomized trial.

### 3.2. Study Characteristics

Supplementary Table S1 presents a detailed summary of the final included reports. Nineteen randomized T2DM reports representing 18 unique trials were retained in the systematic review; 14 reports from 14 unique trials contributed to at least one quantitative synthesis, and five reports were summarized narratively. The comparator interventions included metformin, insulin-based regimens, thiazolidinediones, GLP-1 receptor agonists, DPP-4 inhibitors, SGLT2 inhibitors, alpha-glucosidase inhibitors, and other glucose-lowering strategies. Because these comparators differ substantially in mechanism, expected weight effects, and cardiometabolic profile, we interpret the global pooled estimates as broad summaries of the available randomized evidence rather than direct evidence of equivalence between drug classes.

### 3.3. Risk of Bias Assessment in Individual Studies

**Figure 2:**
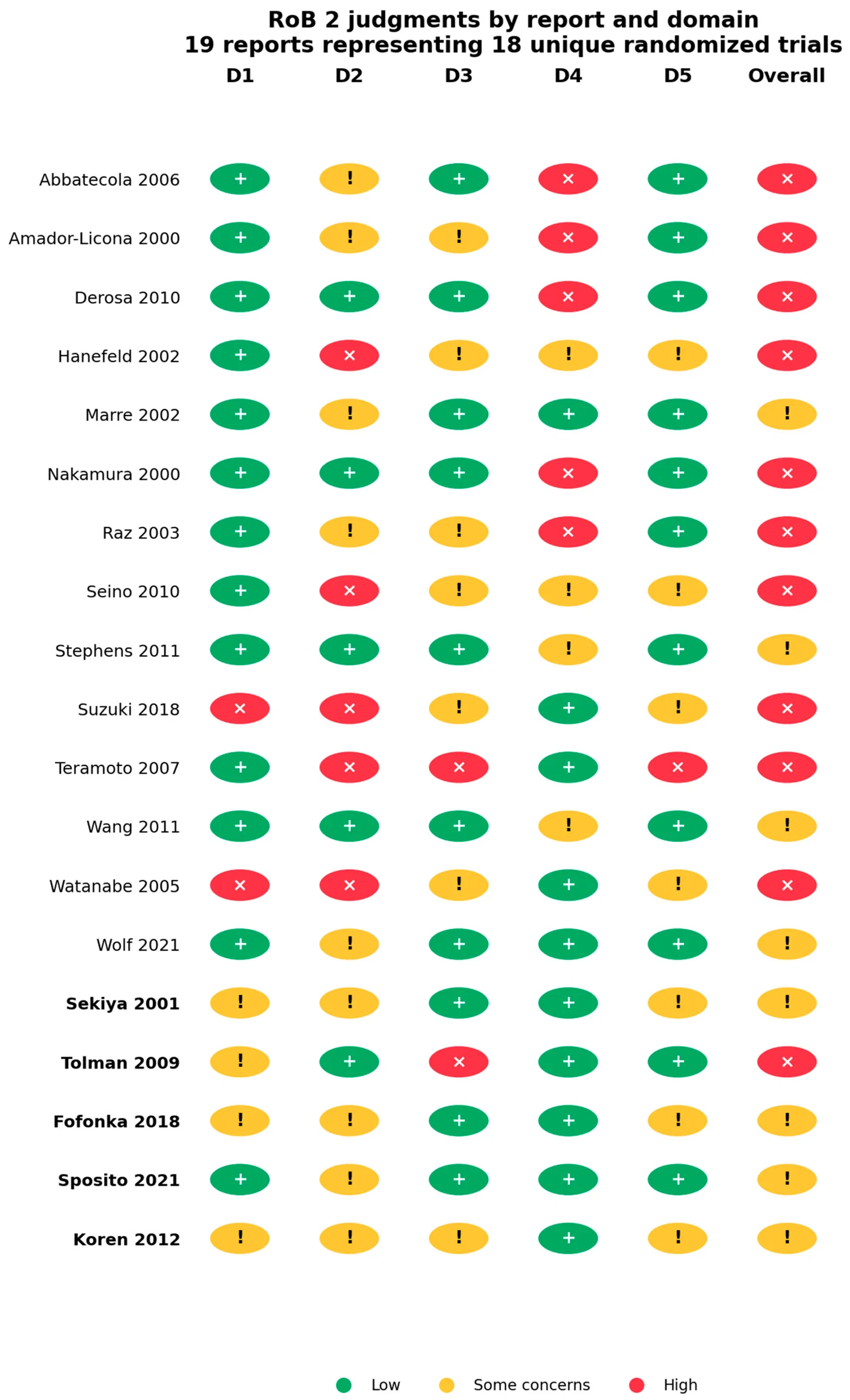
RoB 2 traffic-light plot for a principal eligible result from all 19 included reports (18 unique randomized trials). D1, randomization process; D2, deviations from intended interventions; D3, missing outcome data; D4, measurement of the outcome; D5, selection of the reported result.

**Figure 3:**
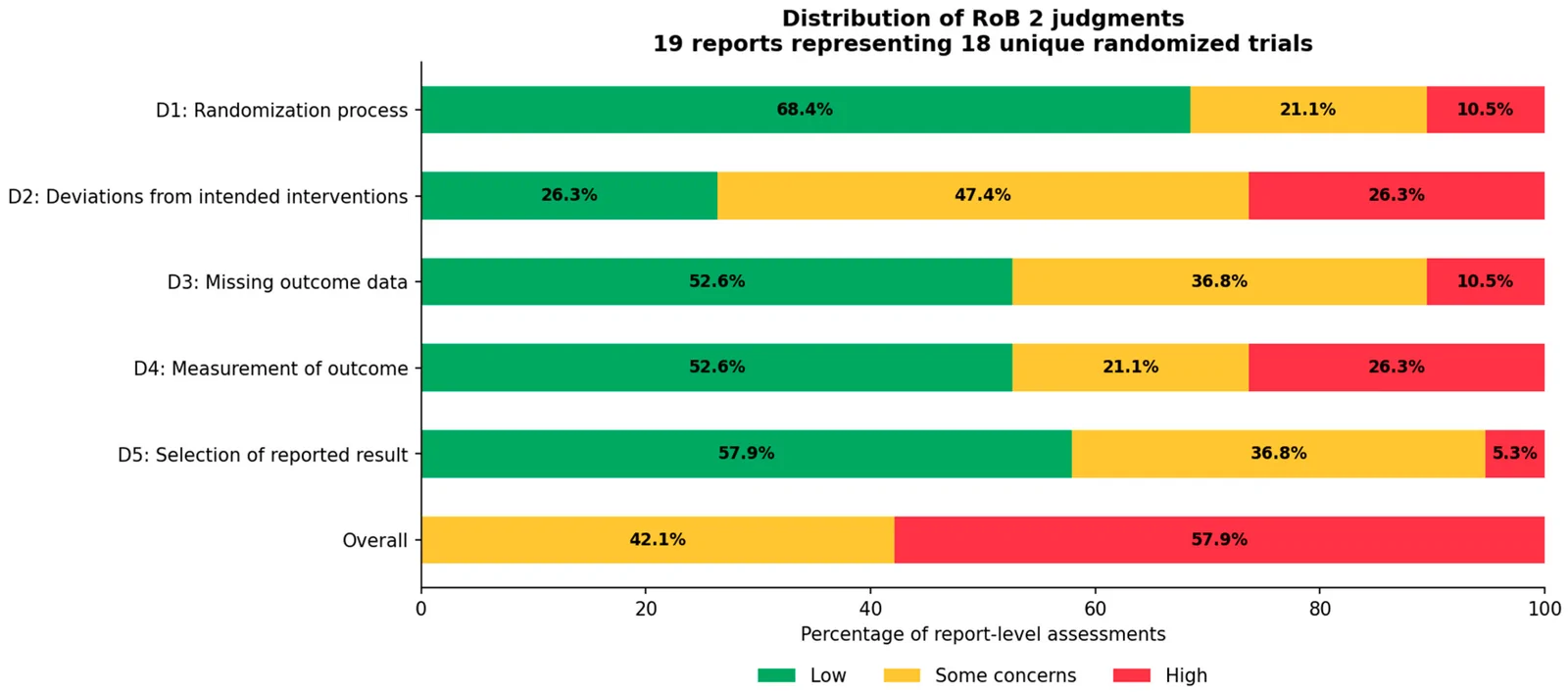
Percentage distribution of RoB 2 judgments across individual domains and overall for the 19 report-level assessments (18 unique randomized trials).

We reassessed risk of bias using RoB 2 for a principal eligible result from all 19 included reports, representing 18 unique randomized trials (Figure 2 and Figure 3). In D1, 13 report-level results (68.4%) were judged at low risk, four (21.1%) raised some concerns, and two (10.5%) were at high risk. In D2, five (26.3%) were at low risk, nine (47.4%) raised some concerns, and five (26.3%) were at high risk. For D3, 10 (52.6%) were at low risk, seven (36.8%) raised some concerns, and two (10.5%) were at high risk. In D4, 10 (52.6%) were at low risk, four (21.1%) raised some concerns, and five (26.3%) were at high risk. In D5, 11 (57.9%) were at low risk, seven (36.8%) raised some concerns, and one (5.3%) was at high risk. Overall, 11 report-level results (57.9%) were classified as high risk and eight (42.1%) as raising some concerns; none met the criteria for an overall low-risk judgment. Within the 14-report quantitative subset, 10 were at high overall risk of bias and four raised some concerns; these quantitative-subset judgments informed the outcome-specific GRADE assessments. We incorporated these limitations into the GRADE judgments and the interpretation of statistically significant results.

### 3.4. Synthesis of Results

The final quantitative synthesis used the same prespecified random-effects framework after re-verification of study design and population eligibility. We pooled continuous outcome data using inverse-variance weighting, using mean differences (MDs) when units were compatible and standardized mean differences (SMDs) only when necessary. Negative estimates indicate lower values with glibenclamide and positive estimates higher values with glibenclamide. Given the marked clinical diversity of comparator therapies, all pooled estimates are interpreted as descriptive, exploratory summaries rather than tests of equivalence between individual treatments.

#### 3.4.1. Outcome Blood Parameters

##### Digital Systolic Blood Pressure (SBP)

**Figure 4:**
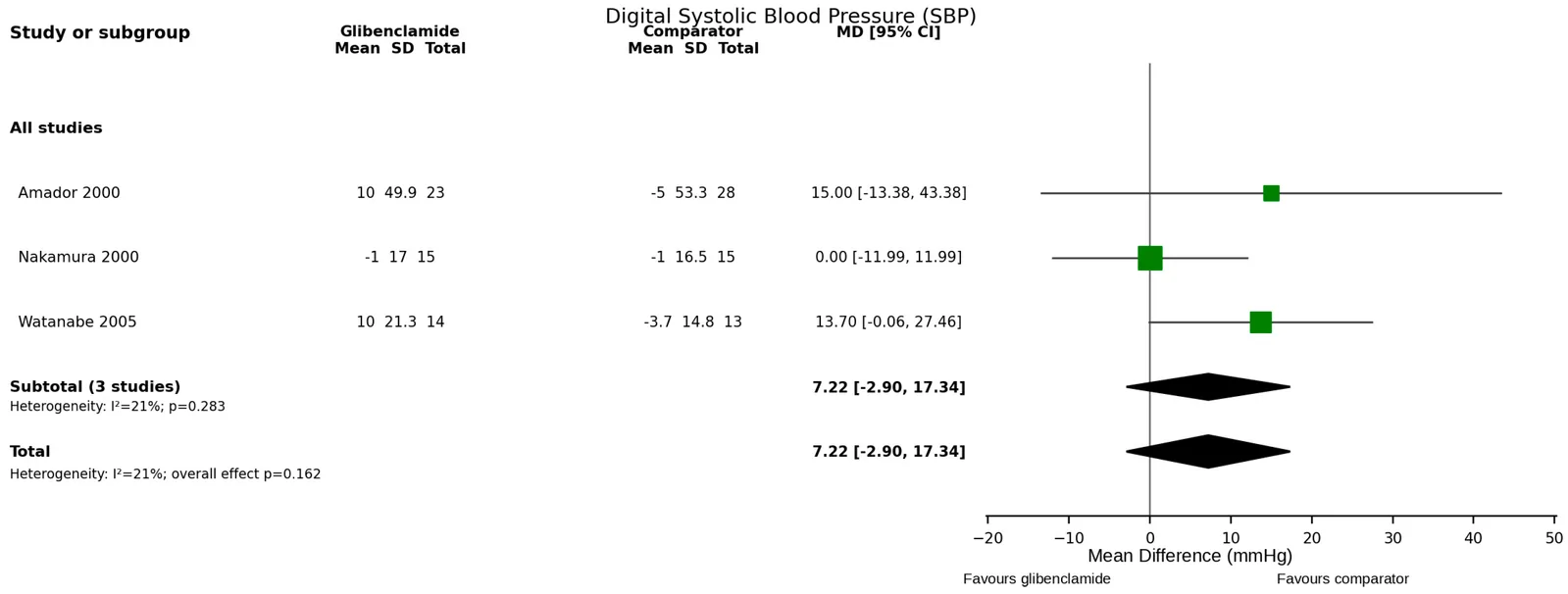
Forest plot: Digital Systolic Blood Pressure (SBP).

Three randomized controlled trials evaluated digital systolic blood pressure (SBP) [11,16,25]. No statistically significant difference was observed between glibenclamide and comparator therapy (MD = 7.22 mmHg; 95% CI −2.90 to 17.34; *p* = 0.16) (Figure 4). Heterogeneity was low to moderate (I^2^ = 21%). Given the small number of trials and the risk-of-bias profile, this estimate remains imprecise and should be interpreted cautiously.

##### Digital Diastolic Blood Pressure (DBP)

**Figure 5:**
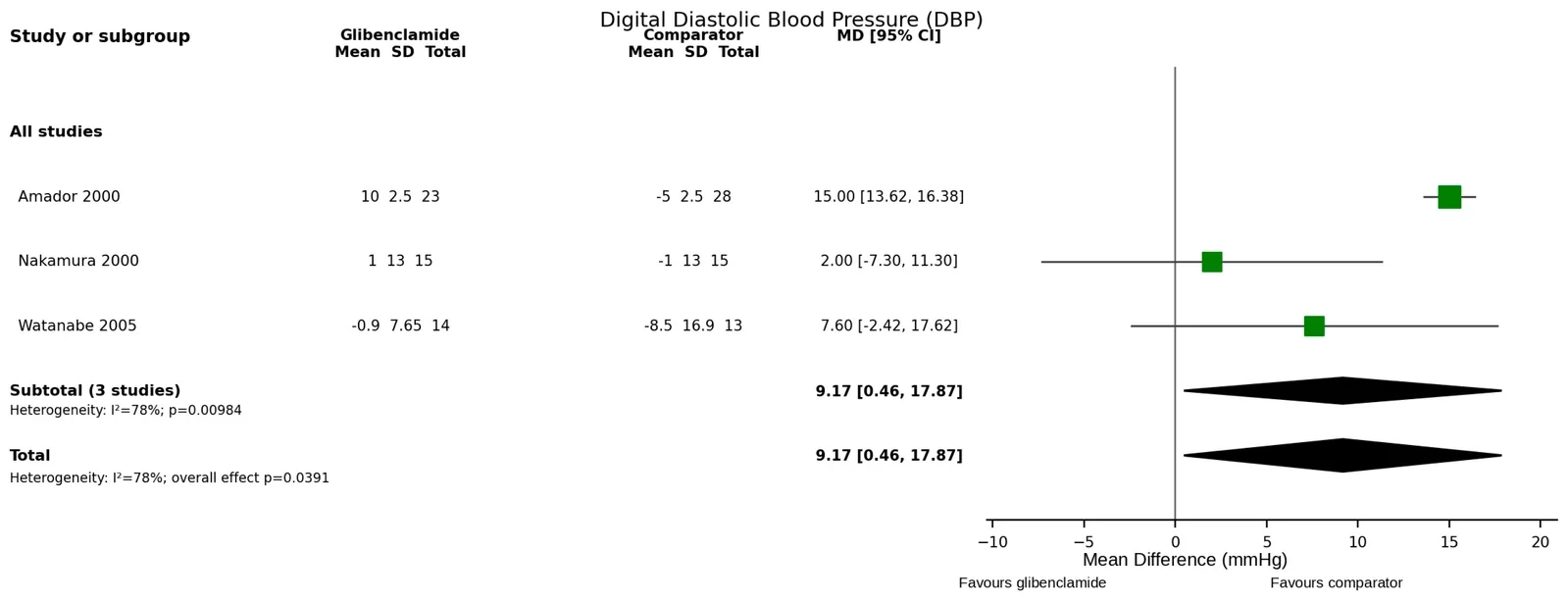
Forest plot Diastolic Blood Pressure.

Three randomized controlled trials evaluated diastolic blood pressure (DBP) [11,16,25]. The pooled estimate was nominally higher with glibenclamide (MD = 9.17 mmHg; 95% CI 0.46 to 17.87; *p* = 0.039) (Figure 5), with substantial heterogeneity (I^2^ = 78%). Because this result is based on only three trials, all with methodological limitations, it should be regarded as low-confidence and hypothesis-generating rather than evidence of a clinically established blood-pressure effect.

##### HbA1c Global

**Figure 6:**
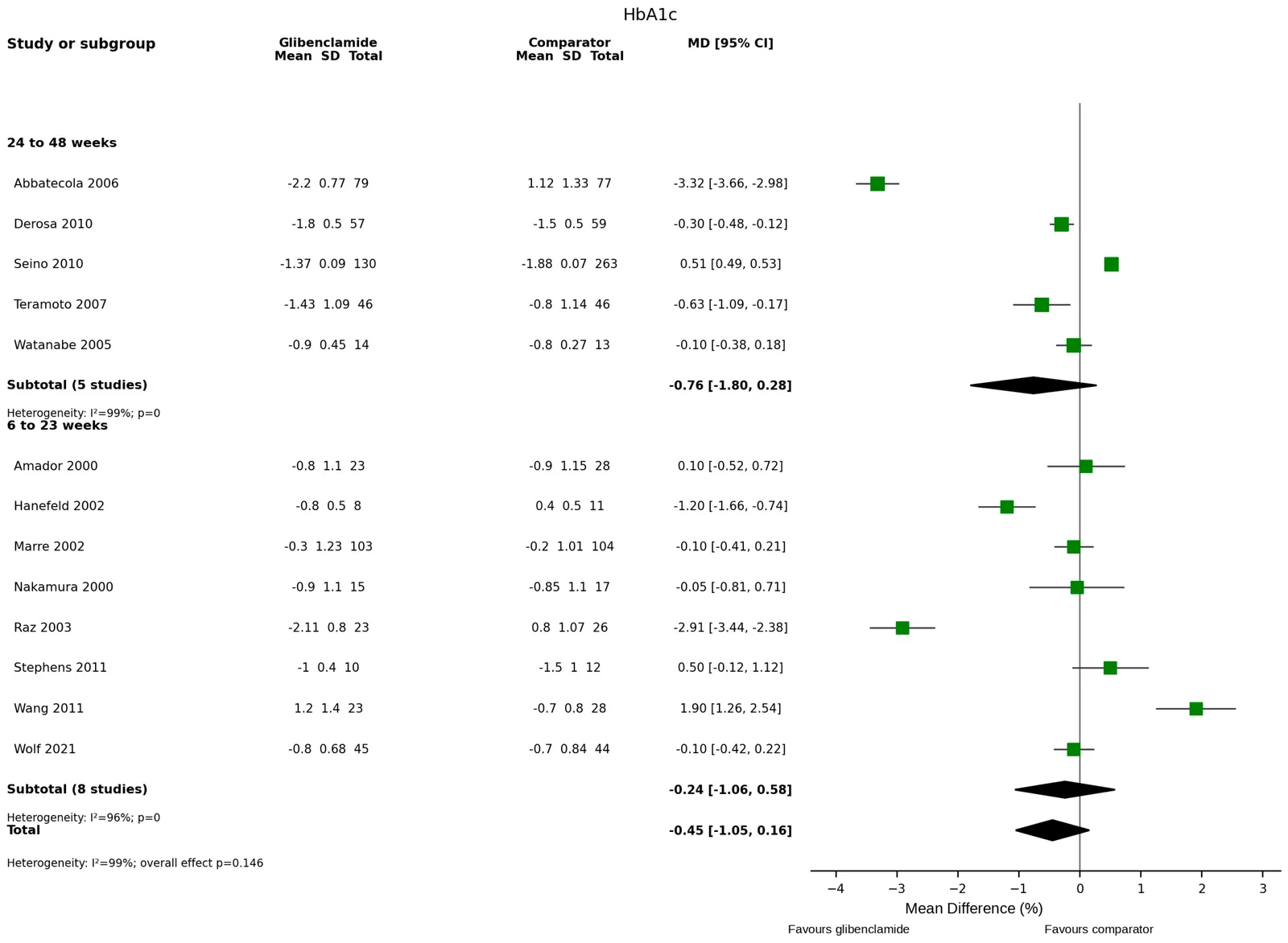
Forest plot HbA1c%.

Thirteen randomized controlled trials assessed HbA1c [9,11,12,13,15,16,17,19,21,23,24,25,26]. No statistically significant difference was observed between glibenclamide and comparator therapies (MD = −0.45%; 95% CI −1.05 to 0.16; *p* = 0.15) (Figure 6). Heterogeneity was extreme (I^2^ = 99%), and the certainty of evidence was downgraded accordingly. The estimate should not be interpreted as evidence of equivalence.

**HbA1c 24 to 48 weeks**

Five randomized controlled trials contributed to the 24–48-week HbA1c subgroup [9,12,19,23,25]. No statistically significant difference was observed (MD = −0.76%; 95% CI −1.80 to 0.28; *p* = 0.15), with very high heterogeneity (I^2^ = 99%).

**HbA1c 6 to 23 weeks**

Eight randomized controlled trials contributed to the 6–23-week HbA1c subgroup [11,13,15,16,17,21,24,26]. No statistically significant difference was observed (MD = −0.24%; 95% CI −1.06 to 0.58; *p* = 0.56), and heterogeneity remained very high (I^2^ = 96%).

##### Fasting Plasma Glucose (FPG) Global

**Figure 7:**
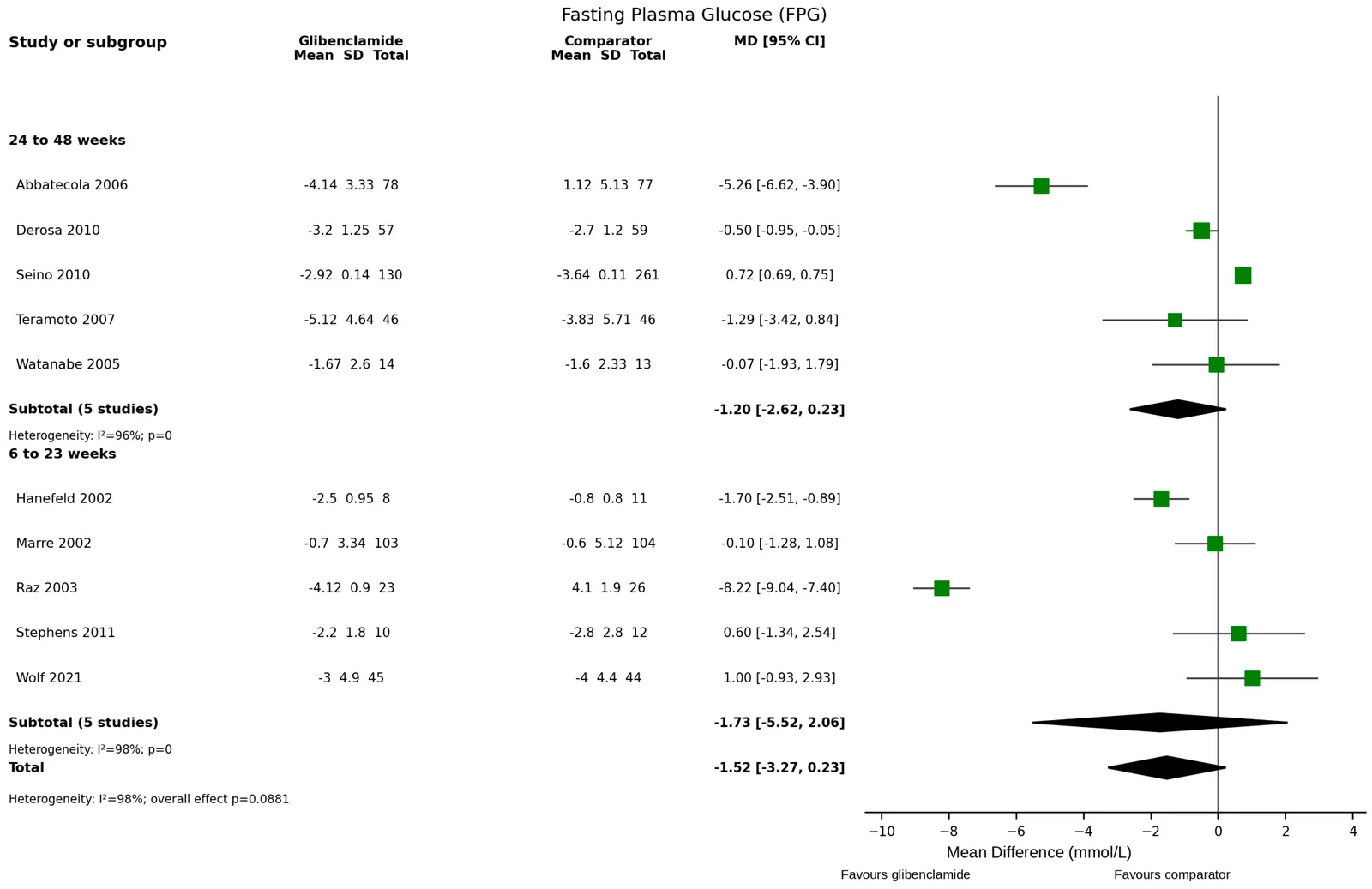
Forest plot: Fasting Plasma Glucose (FPG); all FPG values are expressed in mmol/L.

Ten randomized controlled trials evaluated fasting plasma glucose (FPG) after excluding ineligible studies [9,12,13,15,17,19,21,23,25,26]. The revised forest plot excludes Silva 2007 [20] and, following final design verification, Sarafidis 2005 [18]. No statistically significant difference was observed (MD = −1.52 mmol/L; 95% CI −3.27 to 0.23; *p* = 0.088) (Figure 7). Heterogeneity was extreme (I^2^ = 99%), limiting the precision and clinical interpretability of the pooled estimate.

**Fasting Plasma Glucose (FPG) 24 to 48 Weeks**

Five randomized controlled trials contributed to the 24–48-week FPG subgroup [9,12,19,23,25]. No statistically significant difference was observed (MD = −1.20 mmol/L; 95% CI −2.62 to 0.23; *p* = 0.10), with very high heterogeneity (I^2^ = 96%).

**Fasting Plasma Glucose (FPG) 6 to 23 Weeks**

Five randomized controlled trials contributed to the 6–23-week FPG subgroup [13,15,17,21,26]. No statistically significant difference was observed (MD = −1.73 mmol/L; 95% CI −5.52 to 2.06; *p* = 0.37), with very high heterogeneity (I^2^ = 98%).

##### Fructosamine

**Figure 8:**
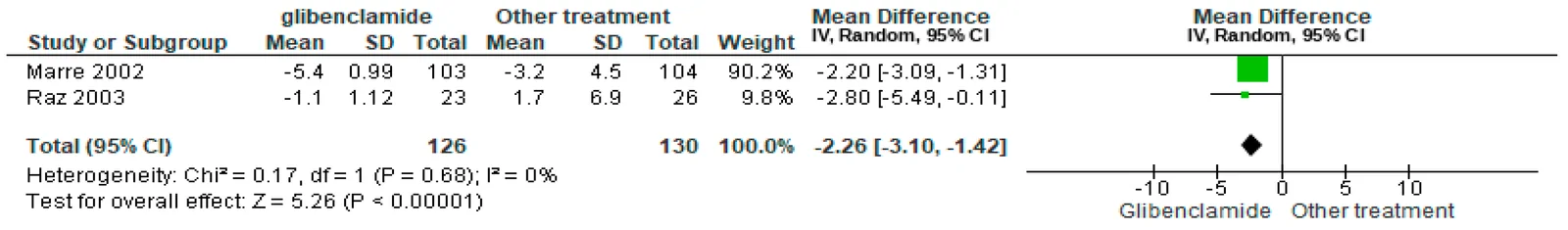
Forest plot fructosamine.

Two randomized controlled trials evaluated fructosamine [15,17]. The pooled analysis favored glibenclamide (MD = −2.26 µmol/L; 95% CI −3.10 to −1.42; *p* < 0.00001) (Figure 8), with no observed statistical heterogeneity (I^2^ = 0%). Because only two studies contributed and certainty was low, this finding should be considered limited evidence of a short-term biochemical difference rather than a definitive clinical benefit. Figure 8 has been corrected to display the prespecified inverse-variance random-effects model; with τ^2^ = 0, the pooled estimate and 95% CI are unchanged.

##### HDL Cholesterol Global

**Figure 9:**
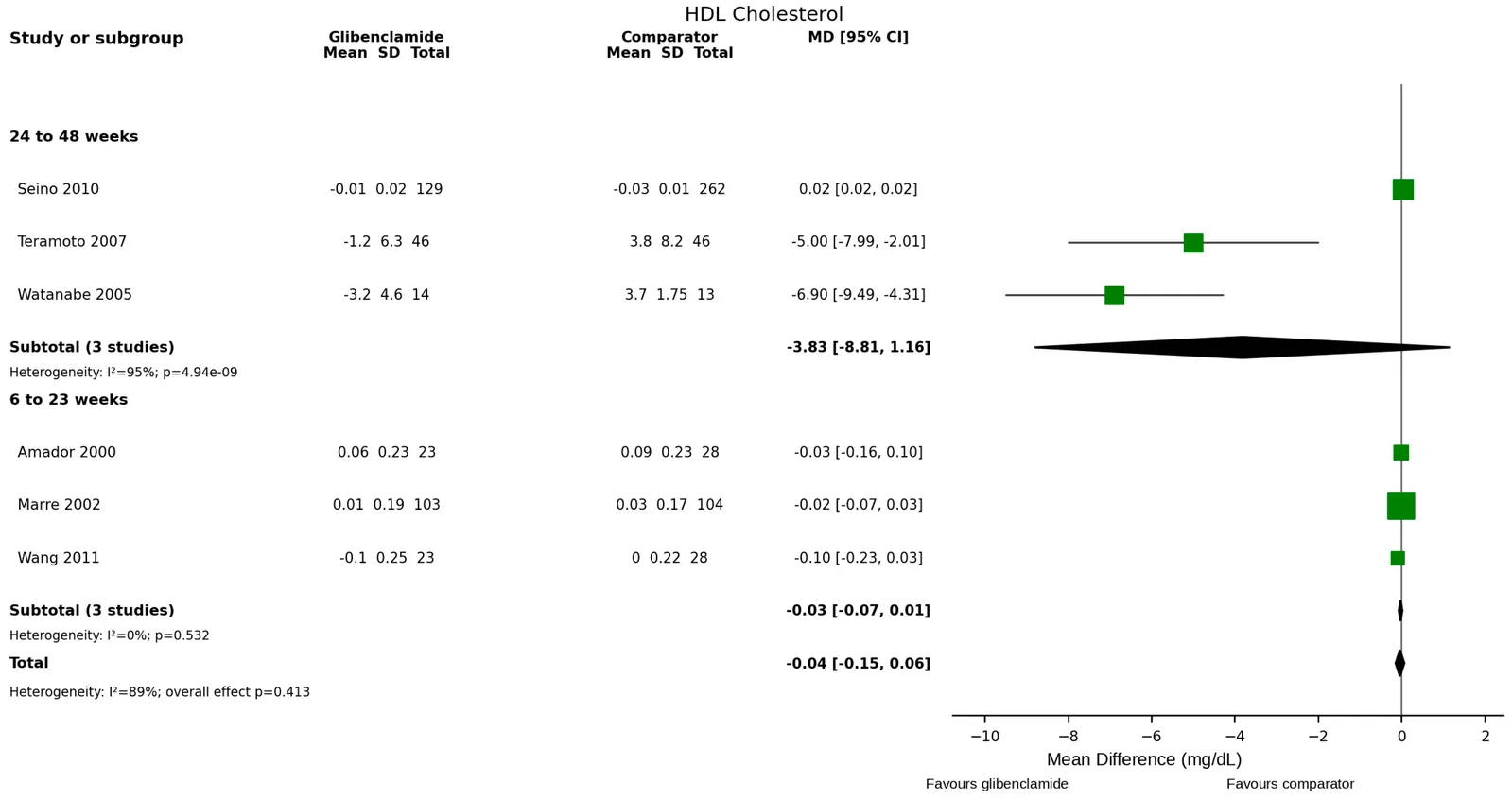
Forest plot HDL-C.

Six randomized controlled trials evaluated HDL-C [11,15,19,23,24,25]. No statistically significant difference was observed (MD = −0.04 mg/dL; 95% CI −0.15 to 0.06; *p* = 0.41) (Figure 9), with substantial heterogeneity (I^2^ = 89%).

**HDL cholesterol 24 to 48 weeks**

Three randomized controlled trials contributed to the 24–48-week HDL-C subgroup [19,23,25]. No significant difference was observed (MD = −3.83 mg/dL; 95% CI −8.81 to 1.16; *p* = 0.13), with very high heterogeneity (I^2^ = 95%).

**HDL Cholesterol 6 to 23 Weeks**

Three randomized controlled trials contributed to the 6–23-week HDL-C subgroup [11,15,24]. No significant difference was observed (MD = −0.03 mg/dL; 95% CI −0.07 to 0.01; *p* = 0.18), with I^2^ = 0%.

##### Low-Density Lipoprotein Cholesterol LDL Global

**Figure 10:**
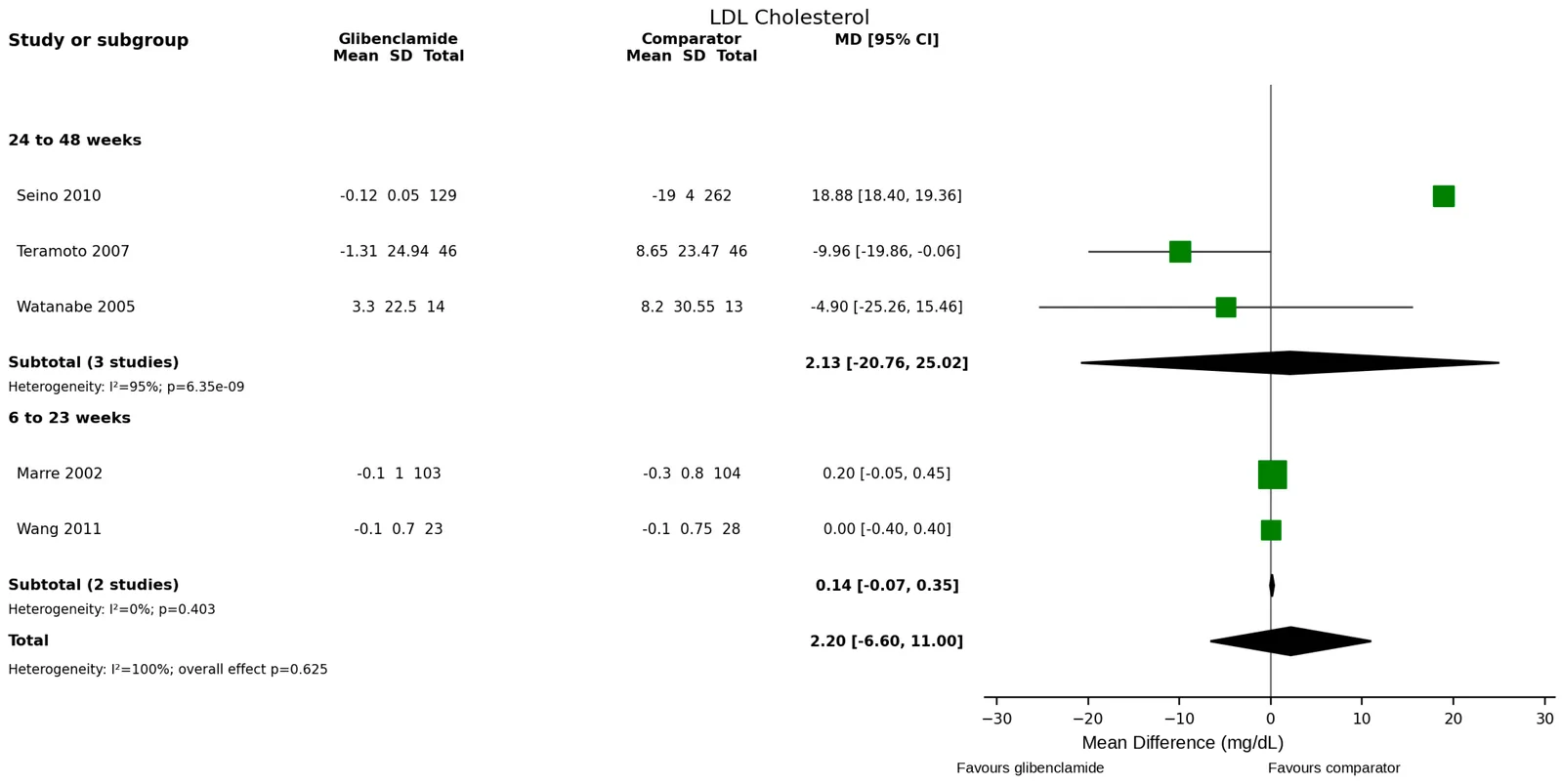
Forest plot LDL-C.

Five randomized controlled trials evaluated LDL-C [15,19,23,24,25]. No significant difference was observed (MD = 2.20 mg/dL; 95% CI −6.61 to 11.00; *p* = 0.62) (Figure 10). Heterogeneity was extreme (I^2^ ≈ 100%), and the estimate is therefore highly uncertain.

**LDL cholesterol 24 to 48 weeks**

Three randomized controlled trials contributed to the 24–48-week LDL-C subgroup [19,23,25]. No statistically significant difference was observed (MD = 2.13 mg/dL; 95% CI −20.76 to 25.02; *p* = 0.86), with very high heterogeneity (I^2^ = 95%).

**LDL Cholesterol 6 to 23 Weeks**

Two randomized controlled trials contributed to the 6–23-week LDL-C subgroup [15,24]. No statistically significant difference was observed (MD = 0.14 mg/dL; 95% CI −0.06 to 0.35; *p* = 0.18), with I^2^ = 0%.

##### Body Mass Index (BMI) Global

**Figure 11:**
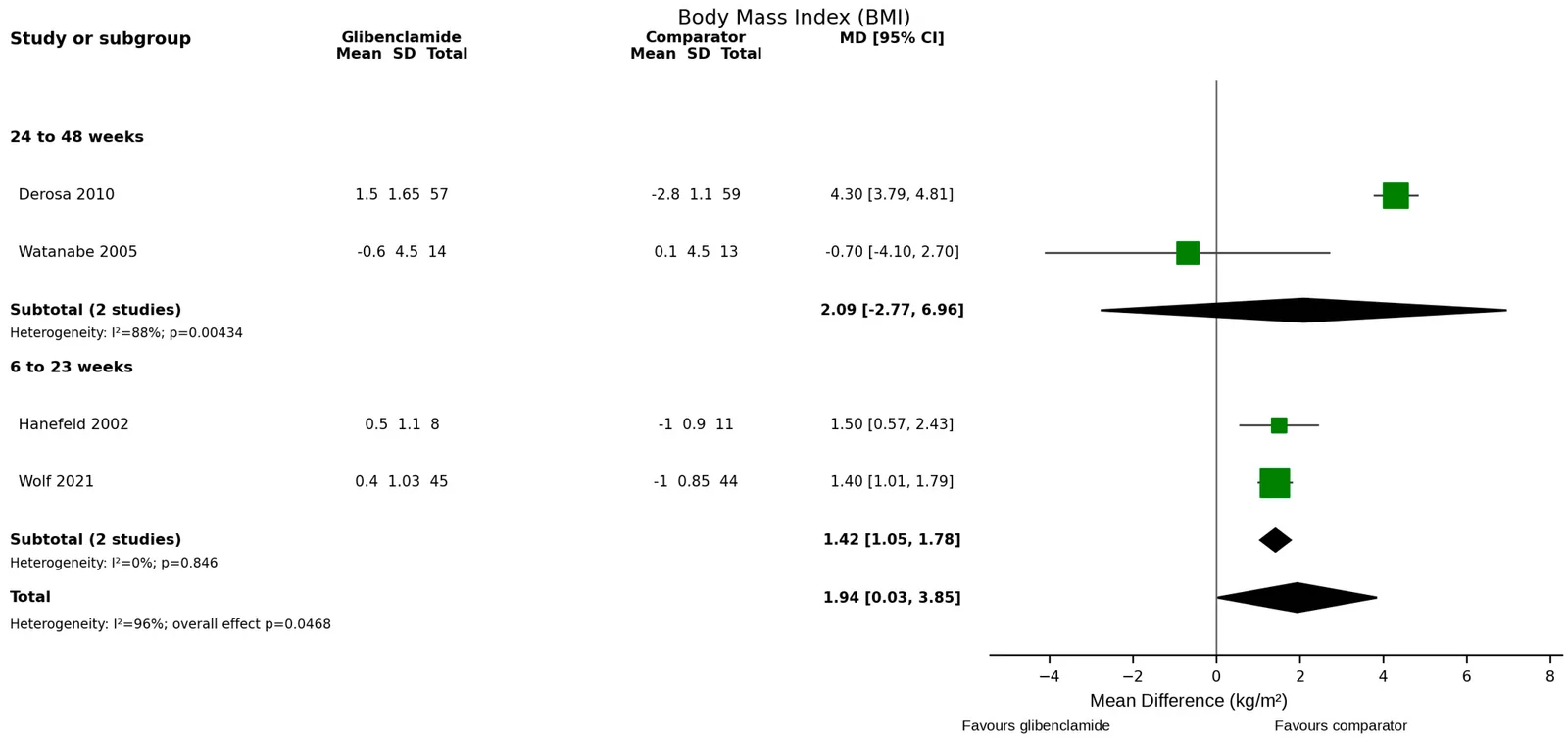
Forest plot for Body Mass Index (BMI).

Four randomized controlled trials evaluated BMI [12,13,25,26]. The pooled estimate was nominally higher with glibenclamide (MD = 1.94 kg/m^2^; 95% CI 0.03 to 3.85; *p* = 0.047) (Figure 11), but heterogeneity was very high (I^2^ = 97%) and certainty was very low. This statistically significant result should therefore be interpreted cautiously and not as a precise estimate of a class-specific treatment effect.

**Body Mass Index (BMI) 24 to 48 weeks**

Two randomized controlled trials contributed to the 24–48-week BMI subgroup [12,25]. No statistically significant difference was observed (MD = 2.09 kg/m^2^; 95% CI −2.77 to 6.96; *p* = 0.40), with I^2^ = 88%.

**Body Mass Index (BMI) 6 to 23 weeks**

Two randomized controlled trials contributed to the 6–23-week BMI subgroup [13,26]. BMI was higher with glibenclamide (MD = 1.42 kg/m^2^; 95% CI 1.05 to 1.78), with I^2^ = 0%; however, the finding is based on only two studies and should be interpreted in the context of overall very low certainty.

##### Fasting Insulin Global

**Figure 12:**
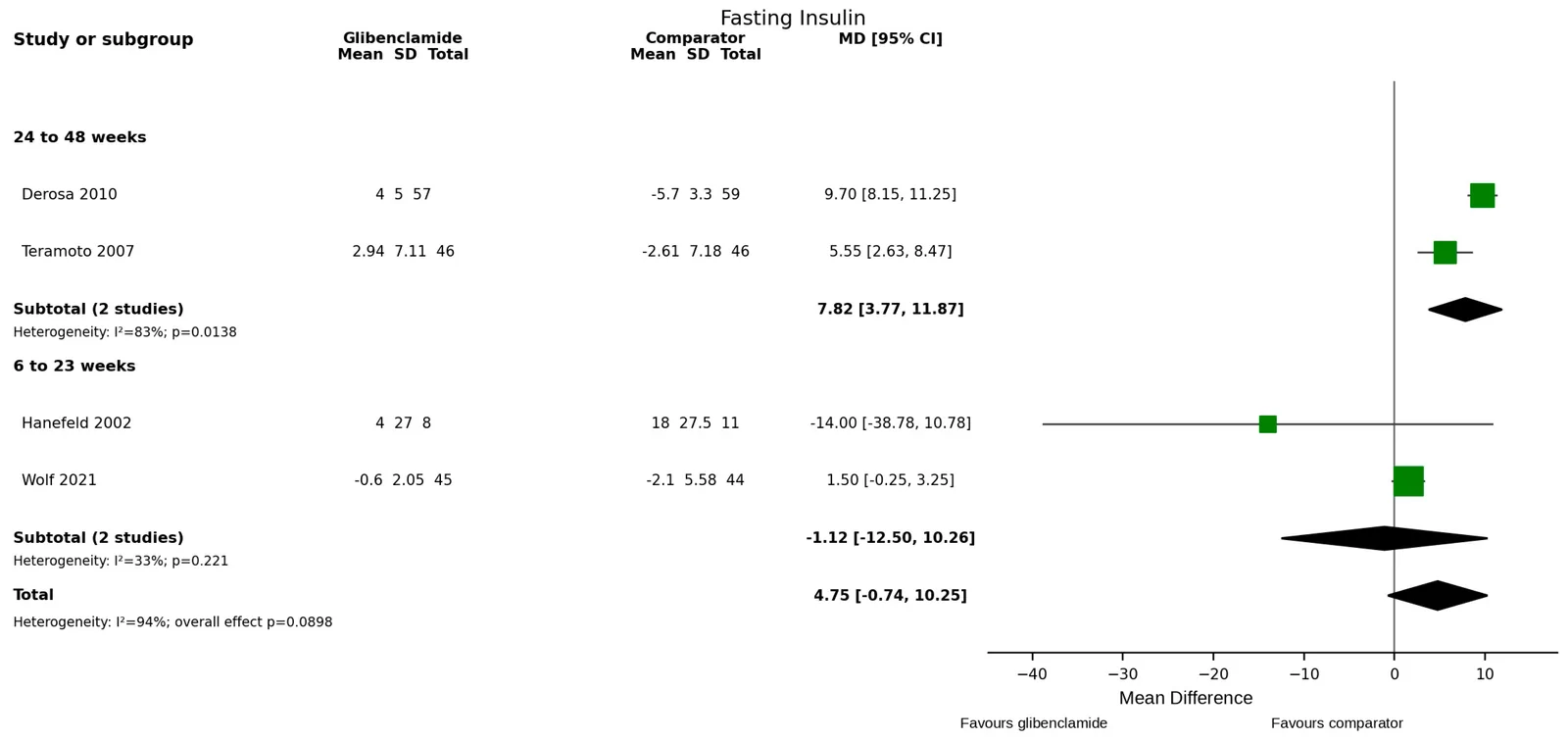
Forest plot for fasting insulin.

Four randomized controlled trials evaluated fasting insulin [12,13,23,26]. Overall, no statistically significant difference was observed (MD = 4.76; 95% CI −0.74 to 10.25; *p* = 0.09) (Figure 12), with very high heterogeneity (I^2^ = 94%).

**Fasting insulin at 24 to 48 weeks**

Two randomized controlled trials contributed to the 24–48-week fasting-insulin subgroup [12,23]. The pooled estimate was higher with glibenclamide (MD = 7.82; 95% CI 3.77 to 11.87), but heterogeneity was substantial (I^2^ = 84%) and the result is based on only two studies; it should therefore be considered exploratory.

**Fasting insulin at 6 to 23 weeks**

Two randomized controlled trials contributed to the 6–23-week fasting-insulin subgroup [13,26]. No statistically significant difference was observed (MD = −1.12; 95% CI −12.50 to 10.27; *p* = 0.85), with I^2^ = 33%.

##### Mean Amplitude of Glycemic Excursions (MAGE)

**Figure 13:**
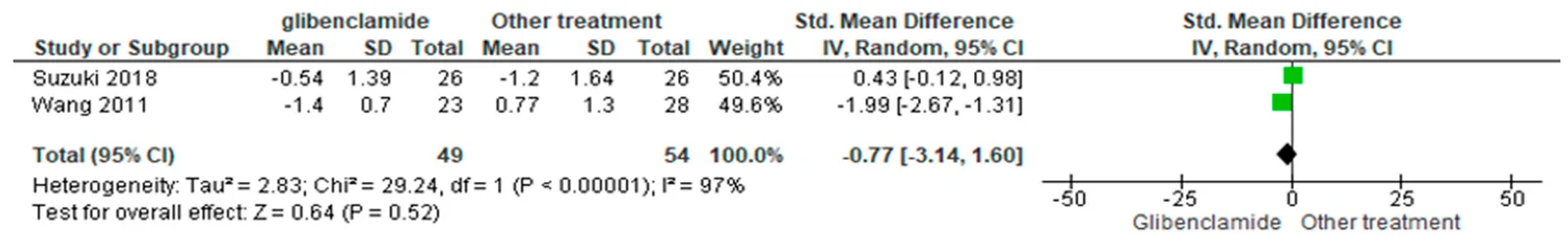
Forest plot for MAGE (Mean Amplitude of Glycemic Excursions).

Two randomized controlled trials evaluated MAGE [22,24]. No statistically significant difference was observed (SMD = −0.77; 95% CI −3.14 to 1.60; *p* = 0.52) (Figure 13). Heterogeneity was very high (I^2^ = 97%), and the very low-certainty estimate should be considered exploratory.

##### C-Reactive Protein (CRP) Global

**Figure 14:**
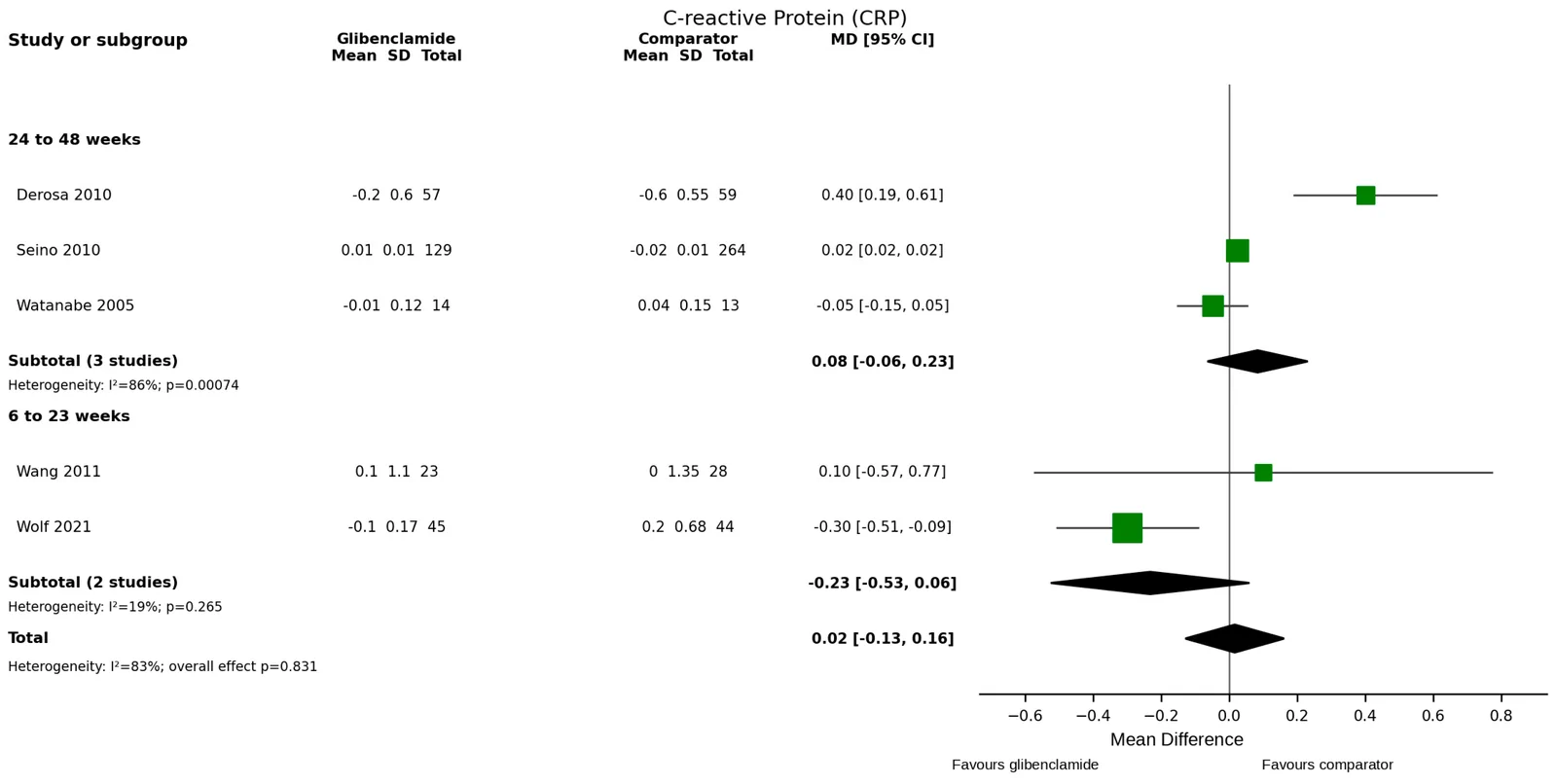
Forest plot CRP (C-reactive protein).

Five randomized controlled trials evaluated CRP [12,19,24,25,26]. No statistically significant difference was observed (MD = 0.02; 95% CI −0.13 to 0.16; *p* = 0.83) (Figure 14), with substantial heterogeneity (I^2^ = 83%). The available trials therefore did not show a consistent effect on CRP; this finding should not be generalized to inflammatory pathways or biomarkers not measured.

**C-reactive protein CRP 24 to 48 weeks**

Three randomized controlled trials contributed to the 24–48-week CRP subgroup [12,19,25]. No statistically significant difference was observed (MD = 0.08; 95% CI −0.07 to 0.23; *p* = 0.27), with I^2^ = 86%.

**C-reactive protein CRP 6 to 23 weeks**

Two randomized controlled trials contributed to the 6–23-week CRP subgroup [24,26]. No statistically significant difference was observed (MD = −0.23; 95% CI −0.53 to 0.06; *p* = 0.12), with I^2^ = 20%.

##### Triglycerides (TG) Global

**Figure 15:**
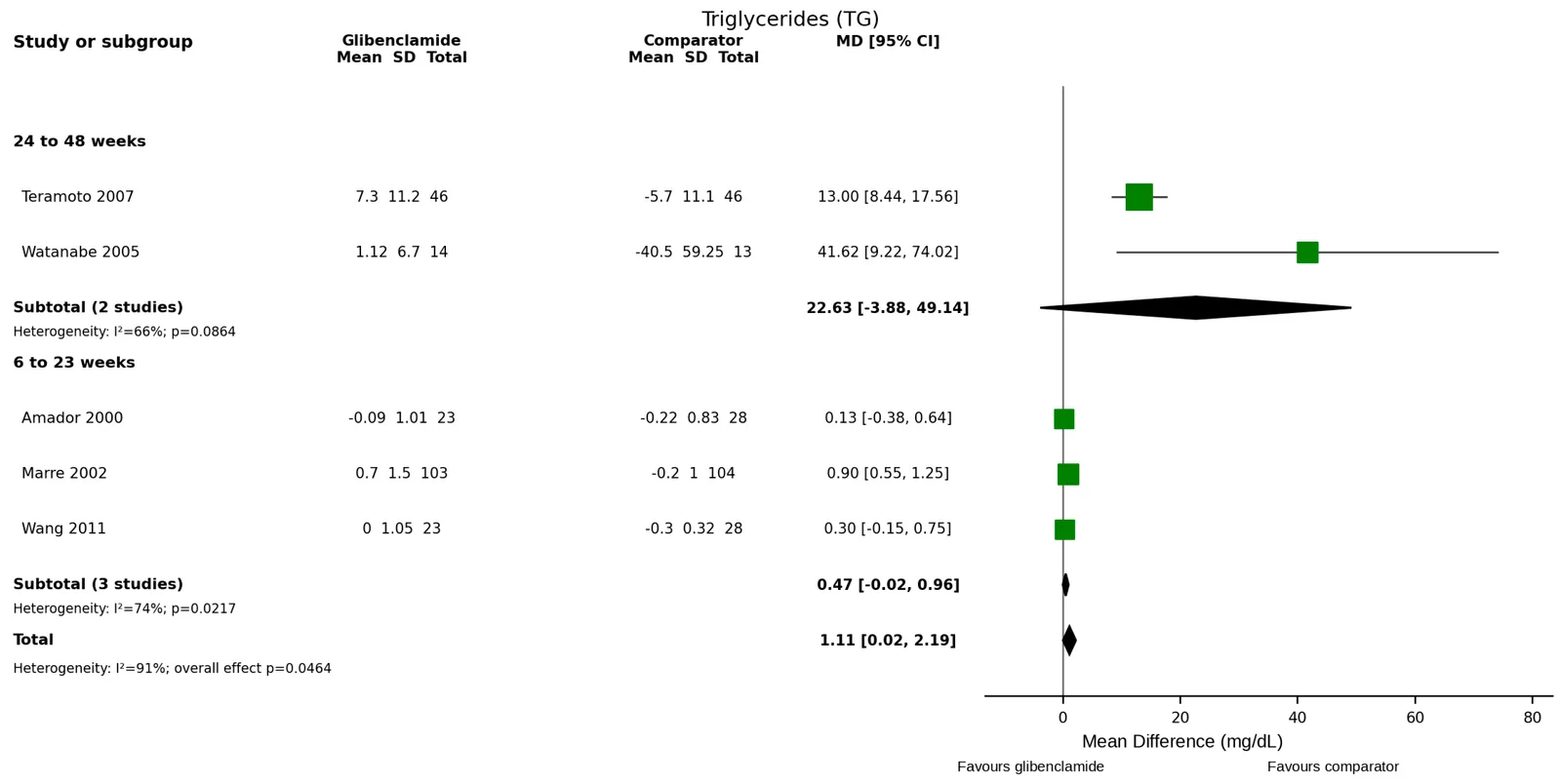
Forest plot for triglycerides (TG).

Five randomized controlled trials evaluated triglycerides [11,15,23,24,25]. The pooled estimate showed a small nominal increase with glibenclamide (MD = 1.11 mg/dL; 95% CI 0.02 to 2.19; *p* = 0.046) (Figure 15), with very high heterogeneity (I^2^ = 91%) and very low certainty. Because statistical significance was not stable across all leave-one-out analyses, the result should be regarded as hypothesis-generating.

**Triglycerides (TG)—24 to 48 weeks**

Two randomized controlled trials contributed to the 24–48-week triglyceride subgroup [23,25]. No statistically significant difference was observed (MD = 22.63 mg/dL; 95% CI −3.88 to 49.14; *p* = 0.09), with I^2^ = 66%.

**Triglycerides (TG) 6 to 23 weeks**

Three randomized controlled trials contributed to the 6–23-week triglyceride subgroup [11,15,24]. No statistically significant difference was observed (MD = 0.47 mg/dL; 95% CI −0.02 to 0.96; *p* = 0.06), with I^2^ = 74%.

##### Body Weight Global

**Figure 16:**
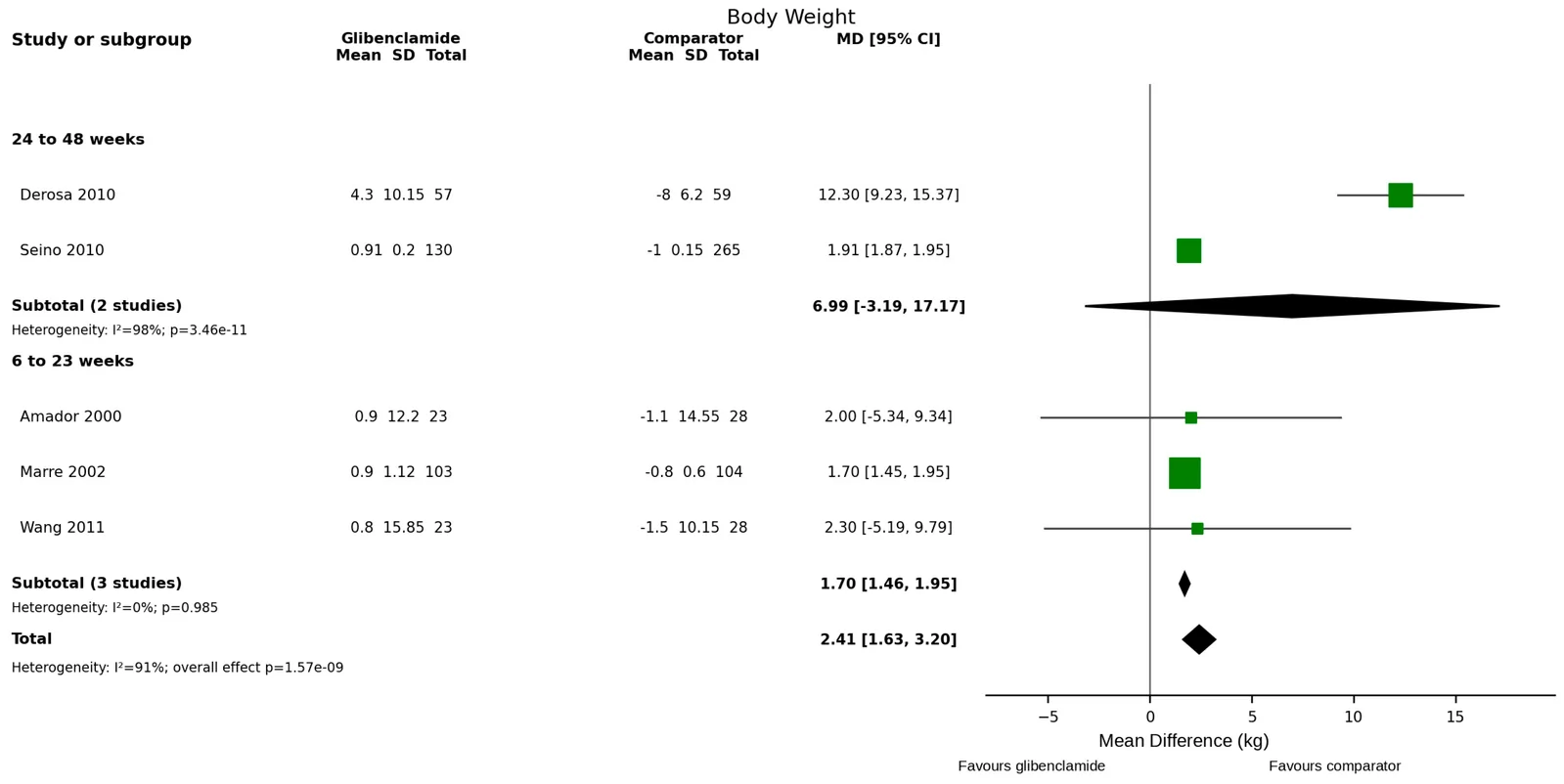
Forest plot for body weight.

Five randomized controlled trials evaluated body weight [11,12,15,19,24]. Body weight was higher with glibenclamide (MD = 2.41 kg; 95% CI 1.63 to 3.20; *p* < 0.001) (Figure 16), with very high heterogeneity (I^2^ = 91%) and very low certainty. The pooled estimate favored comparator therapies, but the magnitude and statistical significance varied in leave-one-out analyses; therefore, interpret the finding cautiously.

**Body Weight 24 to 48 weeks**

Two randomized controlled trials contributed to the 24–48-week body-weight subgroup [12,19]. No significant difference was observed (MD = 6.99 kg; 95% CI −3.19 to 17.17; *p* = 0.18), with I^2^ = 98%.

**Body Weight 6 to 23 weeks**

Three randomized controlled trials contributed to the 6–23-week body-weight subgroup [11,15,24]. Body weight was higher with glibenclamide (MD = 1.70 kg; 95% CI 1.46 to 1.95), with I^2^ = 0%; however, this subgroup is small and should be interpreted cautiously.

##### Total Cholesterol (TC)

**Figure 17:**
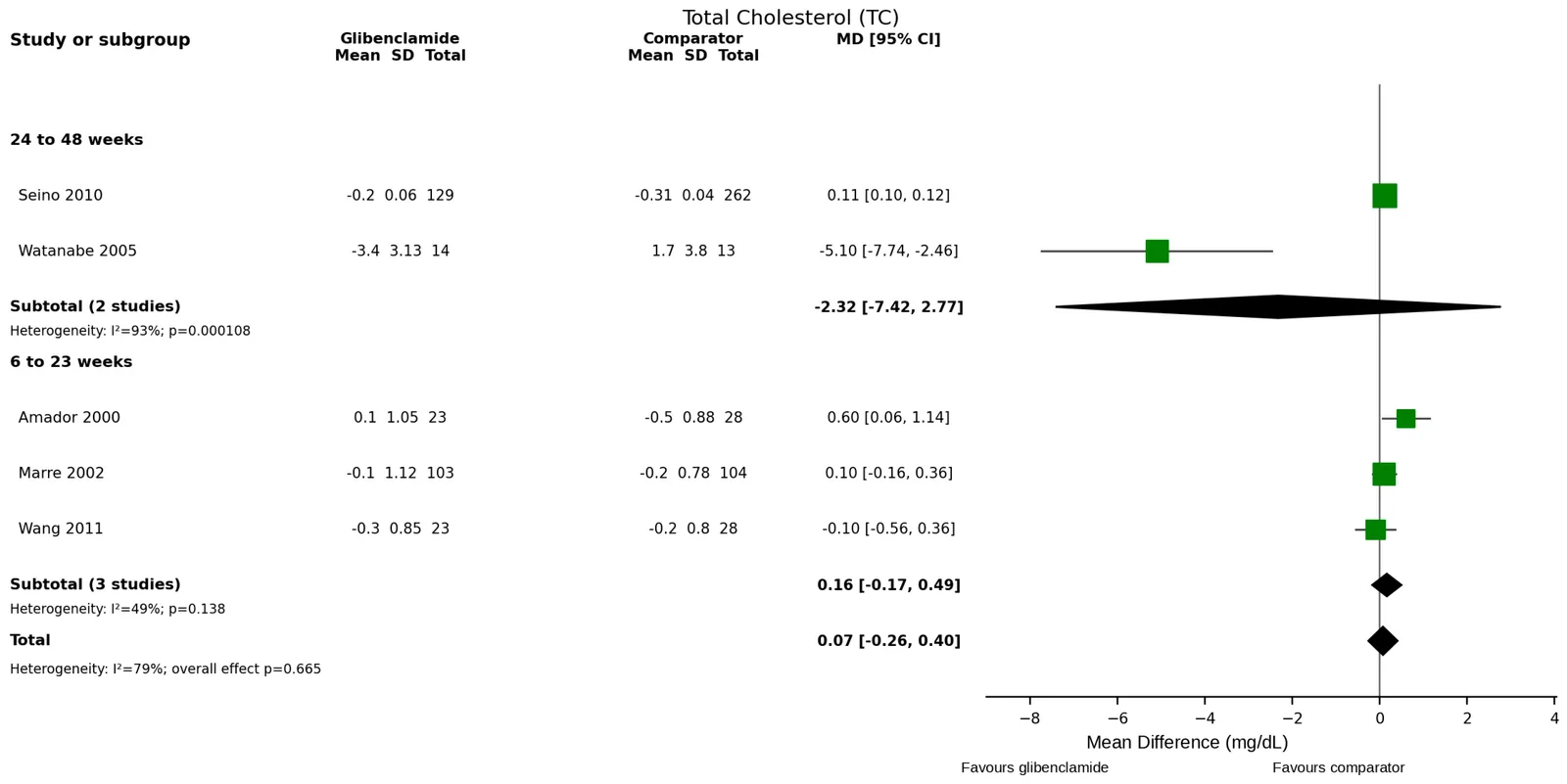
Forest plot for total cholesterol (TC).

Five randomized controlled trials evaluated total cholesterol [11,15,19,24,25]. No statistically significant difference was observed (MD = 0.07 mg/dL; 95% CI −0.26 to 0.40; *p* = 0.66) (Figure 17), with substantial heterogeneity (I^2^ = 79%).

**Total Cholesterol 24 to 48 weeks**

Two randomized controlled trials contributed to the 24–48-week total-cholesterol subgroup [19,25]. No statistically significant difference was observed (MD = −2.32 mg/dL; 95% CI −7.42 to 2.77; *p* = 0.37), with I^2^ = 93%.

**Total Cholesterol 6 to 23 weeks**

Three randomized controlled trials contributed to the 6–23-week total-cholesterol subgroup [11,15,24]. No significant difference was observed (MD = 0.16 mg/dL; 95% CI −0.17 to 0.49; *p* = 0.34), with I^2^ = 50%.

##### Homeostatic Model Assessment of Insulin Resistance (HOMA-IR)

**Figure 18:**
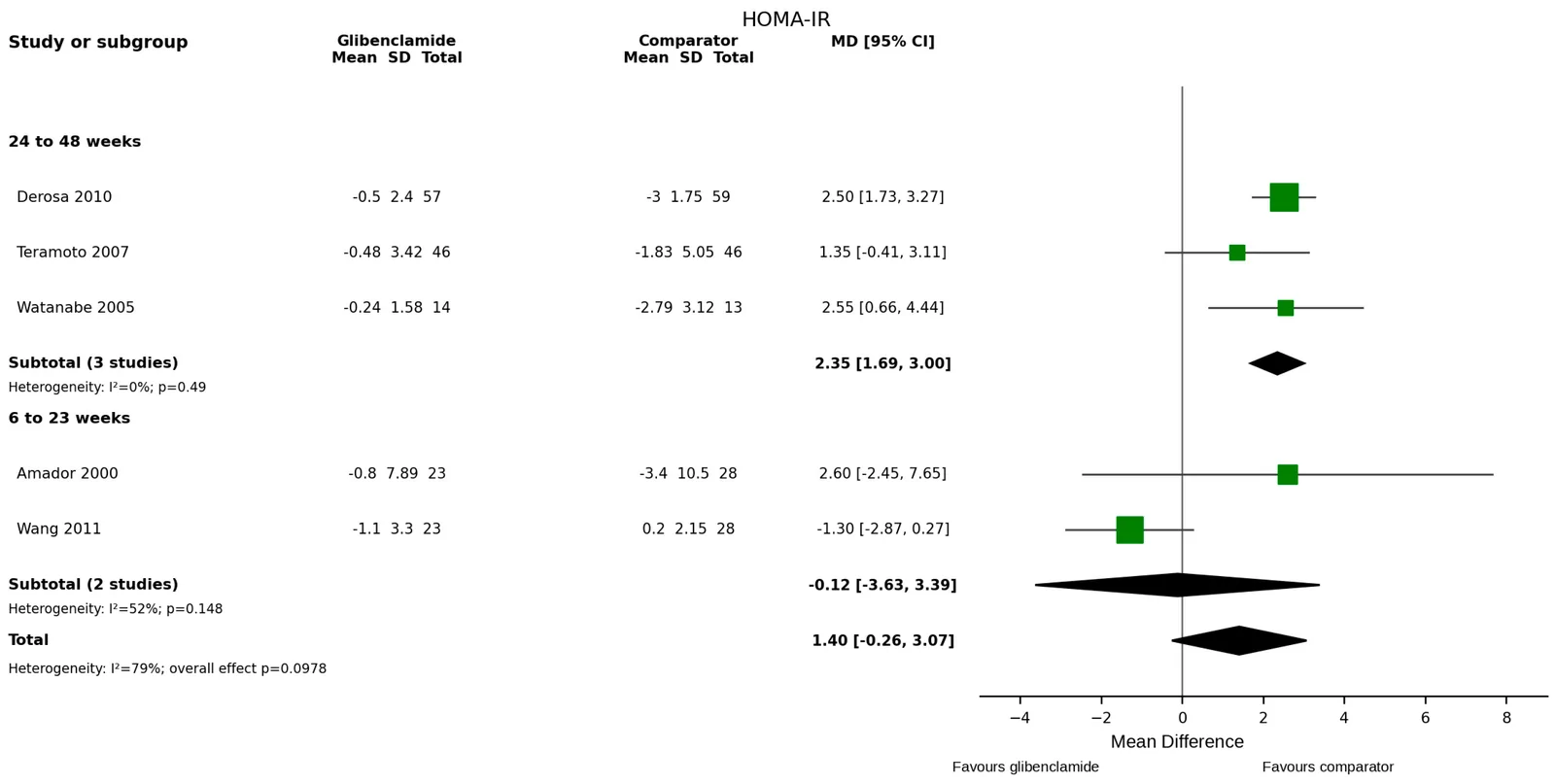
Forest plot for HOMA-IR.

Five randomized controlled trials evaluated HOMA-IR [11,12,23,24,25]. Overall, no statistically significant difference was observed (MD = 1.40; 95% CI −0.26 to 3.07; *p* = 0.10) (Figure 18), with substantial heterogeneity (I^2^ = 79%) and very low certainty.

**HOMA-IR 24 to 48 weeks**

Three randomized controlled trials contributed to the 24–48-week HOMA-IR subgroup [12,23,25]. HOMA-IR was higher with glibenclamide (MD = 2.35; 95% CI 1.69 to 3.00; I^2^ = 0%); however, because only three studies contributed and the overall evidence base has important risk-of-bias limitations, treat this subgroup finding as exploratory.

**HOMA-IR 6 to 23 weeks**

Two randomized controlled trials contributed to the 6–23-week HOMA-IR subgroup [11,24]. No statistically significant difference was observed (MD = −0.12; 95% CI −3.63 to 3.39; *p* = 0.95), with I^2^ = 52%.

**Adverse effects**

Adverse-event reporting was heterogeneous across the randomized T2DM evidence base. Hypoglycemia was the most clinically relevant recurring event, but definitions, ascertainment methods, severity thresholds, and reporting formats differed substantially across trials. Serious and non-serious adverse events were reported inconsistently, and several studies did not provide data suitable for quantitative pooling. Accordingly, we summarized adverse events qualitatively and did not calculate a pooled comparative safety estimate. This limitation is particularly important for glibenclamide because hypoglycemia is a central treatment-related harm.

### 3.5. Sensitivity Analysis

We repeated leave-one-out analyses in the final randomized T2DM dataset. The direction of the pooled estimates was generally stable for HbA1c and FPG, and FPG remained non-significant after exclusion of each individual study. However, nominal statistical significance for several smaller analyses, including DBP, BMI, triglycerides, and body weight, was not preserved after excluding each study individually. These results support a cautious interpretation: the sensitivity analyses do not indicate a single universally dominant study for the major glycemic outcomes, but they also do not justify describing the overall evidence as robust given high heterogeneity, limited study counts, and substantial risk-of-bias concerns.

## 4. Discussion

This systematic review and meta-analysis synthesizes randomized evidence on the comparative metabolic effects of glibenclamide in adults with T2DM after final re-verification of study population and design. We retained 19 reports representing 18 unique randomized trials for the qualitative synthesis, and 14 reports from 14 unique trials for the quantitative synthesis. Across most outcomes, we found no statistically significant between-treatment differences; however, the main message is uncertainty rather than equivalence. Comparator therapies were clinically heterogeneous, statistical heterogeneity was frequently substantial or extreme, and most pooled evidence was of low or very low certainty.

### 4.1. Glycemic Control

Across the principal glycemic endpoints, HbA1c and FPG showed no statistically significant between-treatment differences in the revised RCT-only analyses. Fructosamine favored glibenclamide in two studies, whereas fasting insulin and HOMA-IR showed inconsistent patterns across follow-up strata. Postprandial plasma glucose was removed from the quantitative synthesis after exclusion of the GDM study, leaving only one eligible T2DM RCT. Taken together, these findings should not be described as demonstrating therapeutic comparability: absence of statistical significance, especially with extreme heterogeneity and imprecision, does not establish equivalence.

Fructosamine represents a short-term marker of glycemic control, reflecting average glucose exposure over approximately 2–3 weeks, and may provide complementary information to HbA1c, particularly in clinical scenarios where HbA1c interpretation is limited, such as anemia or conditions affecting erythrocyte turnover [30,31]. In this meta-analysis, the observed reduction in fructosamine with glibenclamide suggests a potential early glycemic effect; however, the limited number of contributing trials and the low certainty of evidence preclude firm conclusions regarding its broader clinical relevance. Elevated fructosamine concentrations have been independently associated with an increased risk of cardiovascular disease, including coronary heart disease, ischemic stroke, heart failure, and all-cause mortality in observational studies [32]. Mechanistically, the reduction in fructosamine observed with glibenclamide could reflect a favorable short-term metabolic signal. Nevertheless, there is currently no direct evidence demonstrating that pharmacologically induced reductions in fructosamine translate into improvements in cardiovascular outcomes or long-term prognosis in patients with type 2 diabetes. Consequently, while fructosamine reduction may reflect early glycemic efficacy, its clinical significance beyond short-term glucose lowering remains uncertain.

### 4.2. Insulin Resistance and Insulin Dynamics

Glibenclamide primarily lowers glucose by stimulating pancreatic insulin secretion through SUR1/KATP-channel signaling; this secretagogue action should be distinguished from insulin sensitivity. Fasting insulin and HOMA-IR are indirect, context-dependent measures and may reflect changes in insulin secretion, hepatic glucose handling, adiposity, and peripheral sensitivity rather than a single mechanism. Human genetic studies involving INPPL1/SHIP2, a regulator of insulin signaling, further illustrate that interindividual variation in insulin-signaling pathways may contribute to heterogeneity in insulin-resistance phenotypes [41,42]. The included trials did not measure such genetic variation, and it is offered only as biological context, not as an explanation established by the present meta-analysis.

### 4.3. Lipid Profile and Cardiovascular Risk Markers

For HDL-C, LDL-C, and total cholesterol, the revised pooled analyses showed no statistically significant between-treatment differences, and heterogeneity was substantial to extreme for several endpoints. Triglycerides showed a small nominal increase with glibenclamide, but this estimate was highly heterogeneous, supported by very low-certainty evidence, and not uniformly stable in sensitivity analysis. Interindividual differences in lipid handling may also contribute to variability in metabolic responses; human data involving ABCA1 and hepatic lipase (LIPC) variants provide relevant biological context [43]. The included trials did not evaluate these pathways, and no causal link between these variants and the treatment effects observed here can be inferred.

### 4.4. Body Weight and Anthropometric Outcomes

Body weight and BMI showed signals toward higher values with glibenclamide in the revised analyses, but the magnitude of these effects varied across studies and certainty was very low. These findings are consistent with the recognized tendency of sulfonylureas to promote weight gain, but direct comparisons with modern therapies should be made cautiously because comparator classes and study eras differ. As contemporary external context, real-world studies of the GLP-1 receptor agonist dulaglutide have reported sustained glycemic improvement and weight reduction, with some cohorts also showing favorable lipid changes [44,45]. These observational data were not part of the present meta-analysis and are cited only to place the historical glibenclamide evidence in the context of current treatment options.

### 4.5. Blood Pressure and Inflammatory Markers

The revised blood-pressure analyses remain uncertain. SBP did not differ significantly, while DBP showed a nominally higher pooled value with glibenclamide based on only three trials and substantial heterogeneity; this should not be interpreted as an established hemodynamic effect. For inflammation, the eligible trials did not demonstrate a consistent effect on CRP. Importantly, a non-significant CRP result does not establish absence of broader anti-inflammatory or pleiotropic effects, because the trials assessed only a limited subset of inflammatory markers and were not designed to characterize inflammatory pathways comprehensively.

### 4.6. Context Within Existing Literature (Comparison with Previous Evidence)

Previous meta-analyses have evaluated glibenclamide in GDM and have focused largely on maternal and neonatal outcomes [27,28,29]. GDM is clinically and pathophysiologically distinct from T2DM. In response to peer review, we excluded GDM studies from the final T2DM evidence set rather than retaining them as an indirect source. We therefore discuss GDM literature only as external context, and it does not contribute to the revised pooled estimates.

### 4.7. Certainty of Evidence and Clinical Implications

A key strength of this review is integrating RoB 2 and GRADE with explicit attention to clinical heterogeneity. RoB 2 was assessed for a principal eligible result from all 19 included reports: 11 report-level results were at high overall risk of bias, and eight raised some concerns; none were at overall low risk. Within the 14-report quantitative subset, 10 were at high overall risk, and four raised some concerns. This substantially limits confidence in nominally significant findings, including those for body weight, BMI, triglycerides, DBP, and selected subgroup results. The revised manuscript therefore avoids equating non-significance with equivalence and treats statistically significant findings supported by very low-certainty evidence as signals requiring confirmation rather than definitive treatment effects.

### 4.8. Limitations and Future Directions

Several limitations warrant emphasis. Comparator therapies differ substantially in mechanism of action, expected effects on body weight and lipids, treatment era, dosing, and follow-up. We retained global random-effects estimates only as broad descriptive summaries because comparator-class subgroup analyses were generally underpowered. Few studies informed many outcomes, and several pooled analyses had very high I^2^ values. After the final restriction to eligible randomized T2DM trials, we removed meta-regression because study counts were too small for stable moderator inference. Formal small-study-effect testing was not performed despite HbA1c and FPG meeting the conventional numerical threshold, because extreme clinical and statistical heterogeneity would make funnel-plot asymmetry difficult to interpret reliably. No included trial set was adequate to establish comparative effects on hard cardiovascular, microvascular, renal, or mortality endpoints. In addition, EMBASE and CENTRAL were not accessible to the review team and therefore were not searched; consequently, relevant eligible studies indexed exclusively in these databases may have been missed.

The RoB 2 findings are an additional major limitation. Across all 19 report-level assessments, 11 were judged at high overall risk of bias, and eight raised some concerns; within the 14-report quantitative subset, 10 were at high overall risk, and four raised some concerns. These limitations may influence individual effect estimates and contribute to inconsistency. Accordingly, do not consider nominal *p*-values in isolation from risk of bias, heterogeneity, and certainty of evidence. Future randomized trials should use rigorous allocation methods, standardized outcome definitions, prespecified analyses, and longer follow-up with clinically important safety and patient-centered outcomes.

Safety outcomes, particularly hypoglycemia, were reported incompletely and heterogeneously. Definitions, ascertainment methods, and presentation formats varied substantially, precluding meaningful quantitative synthesis; conclusions regarding comparative safety should therefore remain cautious. We applied RoB 2 to a principal eligible result from all 19 included reports (18 unique trials), including the five reports summarized only narratively. Because RoB 2 is result-specific, judgments may differ for other outcomes, time points, or separate reports arising from the same underlying trial. The outcome-specific GRADE judgments continue to reflect the studies contributing to each quantitative synthesis and are therefore unchanged by addition of the narrative-only RoB 2 assessments. Future trials should use rigorous randomization and allocation procedures, minimize deviations from assigned interventions, standardize outcome measurement and adverse-event reporting, and provide complete prespecified analyses with longer follow-up for cardiovascular, renal, hypoglycemia, and patient-centered outcomes.

## 5. Conclusions

In adults with T2DM, randomized evidence does not demonstrate consistent between-treatment differences for most metabolic outcomes when glibenclamide is compared with a heterogeneous range of comparator therapies. Interpret signals of higher body weight, BMI, triglycerides, and selected subgroup differences cautiously because they are supported by small study sets, substantial heterogeneity, and low or very low certainty of evidence. These findings do not establish equivalence between drug classes, and comparative safety remains uncertain because hypoglycemia was not reported in a sufficiently standardized manner for quantitative synthesis. This meta-analysis did not evaluate economic cost-effectiveness or healthcare accessibility; therefore, any role of these factors in clinical drug selection is contextual rather than a conclusion derived from the pooled data. Better-designed randomized trials with standardized outcomes and longer follow-up are required.

## Data Availability

All data supporting the reported results are contained within this article and the accompanying Supplementary Material.

## Supplementary Materials

The following supporting information can be downloaded at: https://www.mdpi.com/article/10.3390/life16101754/s1, Table S1. Characteristics of the final included randomized reports (19 reports from 18 unique trials); Table S2. Search strategies; Table S3. Potentially eligible full-text studies excluded and reasons; Table S4. Summary of Findings and GRADE (final RCT-only quantitative set); Table S5. RoB 2 judgments for a principal eligible result from all included reports.
