# Supplementary material for: Comparative Metabolic Effects of Glibenclamide in Type 2 Diabetes Mellitus: A Systematic Review and Meta-Analysis of Randomized Controlled Trials
Source: Life (Basel). 2026 Oct 20;16(10):1754. doi: 10.3390/life16101754 (PMC13705965; doi:10.3390/life16101754)
Supplement: Supplementary file 1 [file life-16-01754-s001.zip › life-4501034-supplementary.pdf]

Supplementary Tables – Revised Final Eligibility Set

Manuscript: Comparative Metabolic Effects of Glibenclamide in Type 2 Diabetes Mellitus: A Systematic Review and Meta-Analysis of Randomized Controlled Trials

The supplementary material below reflects the final RCT-only T2DM eligibility set after the design and population re-verification requested during peer review. The final restriction to randomized controlled trials in adults with T2DM represents an eligibility refinement introduced during peer-review re-verification to align the final evidence set with the review title and objective. **The final systematic-review set contains 19 reports representing 18 unique randomized trials.**

Supplementary Table S1. Characteristics of the final included randomized **reports (19 reports from 18 unique trials)**

Study	Design	Population	Comparator	Quantitative synthesis
Abbatecola et al., 2006	Parallel RCT	Adults with T2DM	Repaglinide / comparative glucose-lowering regimen	Yes
Amador-Licona et al., 2000	Randomized comparative trial	Adults with T2DM	Metformin	Yes
Derosa et al., 2010	Parallel RCT	Adults with T2DM	Exenatide	Yes
Hanefeld et al., 2002	Randomized comparative trial	Adults with T2DM	Acarbose	Yes
Marre et al., 2002	Randomized comparative trial	Adults with T2DM	Metformin-based regimen	Yes
Nakamura et al., 2000	Randomized comparative trial	Adults with T2DM	Pioglitazone / voglibose	Yes
Raz et al., 2003	Randomized comparative trial	Adults with T2DM	Insulin-based regimen with rosiglitazone	Yes
Seino et al., 2010	Randomized comparative trial	Adults with T2DM	Liraglutide	Yes
Stephens et al., 2011	Randomized comparative trial	Adults with T2DM	Repaglinide	Yes
Suzuki et al., 2018	Randomized trial	Adults with T2DM	Sitagliptin	Yes
Teramoto et al., 2007	Randomized comparative trial	Adults with T2DM	Pioglitazone	Yes
Wang et al., 2011	Randomized open-label parallel-group trial	Adults with T2DM	Acarbose	Yes
Watanabe et al., 2005	Randomized comparative trial	Adults with T2DM	Pioglitazone	Yes
Wolf et al., 2021	Randomized trial	Adults with T2DM	Dapagliflozin	Yes
Sekiya et al., 2001	Randomized trial	Adults with T2DM/NIDDM	Troglitazone	No – outcome/reporting not compatible with final pooled endpoints
Tolman et al., 2009	Randomized double-blind comparator-controlled trial	Adults with T2DM	Pioglitazone	No – primarily hepatic safety endpoint
Fofonka et al., 2018	Randomized controlled trial	Adults with T2DM	Vildagliptin	No – exercise-specific outcome structure not compatible with pooled endpoint/timepoint
Sposito et al., 2021	Randomized active-controlled trial	Adults with T2DM and atherosclerotic disease	Dapagliflozin	No – vascular primary endpoints / incompatible extraction for final pooled set
Koren et al., 2012	Randomized crossover trial	Adults with T2DM	Sitagliptin	No – crossover report did not provide a paired effect estimate compatible with the prespecified parallel-group pooling method

Note: Final inclusion required randomized allocation and a T2DM population. GDM and non-randomized studies were removed. **The 19 included reports represent 18 unique randomized trials. Wolf et al. (2021) and Sposito et al. (2021) are separate publications from the ADDENDA-BHS2 trial (NCT02919345); Wolf et al. contributed to quantitative synthesis, whereas Sposito et al. was summarized narratively. The quantitative subset contains 14 reports from 14 unique trials.**

Supplementary Table S2. Search strategies

Database	Search strategy	Results (15 Feb 2026)
MEDLINE/PubMed	glibenclamide OR glyburide AND diabetes mellitus NOT animals NOT review; filters: Clinical Trial, Randomized Controlled Trial	603
Web of Science	glibenclamide OR glyburide AND diabetes mellitus NOT animals NOT review	49
CINAHL	glibenclamide OR glyburide AND diabetes mellitus NOT animals NOT review; clinical-trial/RCT publication filters when available	51
Scopus	glibenclamide OR glyburide AND diabetes mellitus NOT animals NOT review	40
Google Scholar	glibenclamide OR glyburide AND diabetes mellitus NOT animals NOT review	81

All searches were last updated on 15 February 2026. This table reports the databases and strategies documented in the supplied search record; no database search has been retrospectively claimed without a corresponding search record. EMBASE and CENTRAL were not searched because these databases were not accessible to the review team; this limitation is acknowledged in the main manuscript.

Supplementary Table S3. Potentially eligible full-text studies excluded and reasons

No.	Reference	Reason for exclusion
1	UKPDS 33, 1998	Glibenclamide-specific outcome data were not separately extractable from the broader sulfonylurea/insulin strategy comparison.
2	Shuster et al., 2020	Gestational diabetes mellitus; population outside the final T2DM scope.
3	Langer et al., 2000	Gestational diabetes mellitus; population outside the final T2DM scope.
4	Silva et al., 2012	Gestational diabetes mellitus; population outside the final T2DM scope.
5	Mirzamoradi et al., 2015	Gestational diabetes mellitus; population outside the final T2DM scope.
6	Manitpisitkul et al., 2013	Pharmacokinetic drug–drug interaction study without an eligible comparative metabolic outcome set.
7	Beltrand et al., 2019	Neonatal diabetes; population outside the final T2DM scope.
8	Abbink et al., 2002	Did not meet the final randomized T2DM treatment-comparison eligibility criterion; removed from the RCT-only quantitative synthesis.
9	Harada et al., 2006	Random allocation was not sufficiently documented for the final RCT-only criterion; removed after design re-verification.
10	Sarafidis et al., 2005	Open-label observational study; non-randomized design.
11	Silva et al., 2007	Gestational diabetes mellitus; removed from the primary T2DM synthesis.
12	Anjalakshi et al., 2007	Gestational diabetes mellitus; removed from the final T2DM evidence set.
13	Kadoi et al., 2002	Insulin-dependent surgical population and no eligible randomized glibenclamide treatment contrast.
14	Mirmiranpour et al., 2016	Randomized L-lysine supplementation on background metformin/glibenclamide; glibenclamide was not the randomized treatment contrast of interest.

Supplementary Table S4. Summary of Findings and GRADE (final RCT-only quantitative set)

Outcome	k	N glib.	N comp.	Effect (95% CI)	I²	Risk of bias	Inconsistency	Imprecision	Certainty
Systolic blood pressure	3	52	56	MD 7.22 (-2.90 to 17.34)	21%	Serious	Not serious	Serious	Low
Diastolic blood pressure	3	52	56	MD 9.17 (0.46 to 17.87)	78%	Serious	Very serious	Serious	Very low
HbA1c	13	576	728	MD -0.45 (-1.05 to 0.16)	99%	Serious	Very serious	Serious	Very low

Outcome	k	N glib.	N comp.	Effect (95% CI)	I²	Risk of bias	Inconsistency	Imprecision	Certainty
Fasting plasma glucose	10	514	653	**MD -1.52 (-3.27 to 0.23) mmol/L**	99%	Serious	Very serious	Serious	Very low
Fructosamine	2	126	130	**MD -2.26 (-3.10 to -1.42) μmol/L**	0%	Serious	Not serious	Serious	Low
HDL-C	6	338	481	MD -0.04 (-0.15 to 0.06)	89%	Serious	Very serious	Serious	Very low
LDL-C	5	315	453	MD 2.20 (-6.61 to 11.00)	~100%	Serious	Very serious	Serious	Very low
Body mass index	4	124	127	MD 1.94 (0.03 to 3.85)	97%	Serious	Very serious	Serious	Very low
Fasting insulin	4	156	160	MD 4.76 (-0.74 to 10.25)	94%	Serious	Very serious	Serious	Very low
MAGE	2	49	54	SMD -0.77 (-3.14 to 1.60)	97%	Serious	Very serious	Serious	Very low
C-reactive protein	5	268	408	MD 0.02 (-0.13 to 0.16)	83%	Serious	Very serious	Serious	Very low
Triglycerides	5	209	219	MD 1.11 (0.02 to 2.19)	91%	Serious	Very serious	Serious	Very low
Body weight	5	336	484	MD 2.41 (1.63 to 3.20)	91%	Serious	Very serious	Serious	Very low
Total cholesterol	5	292	435	MD 0.07 (-0.26 to 0.40)	79%	Serious	Very serious	Serious	Very low
HOMA-IR	5	163	174	MD 1.40 (-0.26 to 3.07)	79%	Serious	Very serious	Serious	Very low

GRADE judgments were updated to reflect the final RCT-only quantitative dataset. Because most outcomes were informed by studies with important RoB 2 concerns, substantial or extreme heterogeneity, and/or wide confidence intervals, certainty was generally very low. We did not grade PPG and ApoB as pooled outcomes because only one eligible randomized T2DM study remained for each after final verification. Indirectness was not downgraded for the broad comparator question, and no additional upgrading or downgrading considerations were applied beyond the domains shown. Publication bias could not be formally assessed and was not used as an additional downgrade. **The newly added RoB 2 assessments for reports retained only in narrative synthesis do not alter the outcome-specific GRADE ratings in this table, which are based on the evidence contributing to each pooled outcome.**

Supplementary Table S5. RoB 2 judgments for a principal eligible result from all included reports

Report	D1	D2	D3	D4	D5	Overall
Abbatecola 2006	Low	Some concerns	Low	High	Low	High
Amador-Licona 2000	Low	Some concerns	Some concerns	High	Low	High
Derosa 2010	Low	Low	Low	High	Low	High
Hanefeld 2002	Low	High	Some concerns	Some concerns	Some concerns	High
Marre 2002	Low	Some concerns	Low	Low	Low	Some concerns
Nakamura 2000	Low	Low	Low	High	Low	High
Raz 2003	Low	Some concerns	Some concerns	High	Low	High
Seino 2010	Low	High	Some concerns	Some concerns	Some concerns	High
Stephens 2011	Low	Low	Low	Some concerns	Low	Some concerns
Suzuki 2018	High	High	Some concerns	Low	Some concerns	High
Teramoto 2007	Low	High	High	Low	High	High
Wang 2011	Low	Low	Low	Some concerns	Low	Some concerns
Watanabe 2005	High	High	Some concerns	Low	Some concerns	High
Wolf 2021	Low	Some concerns	Low	Low	Low	Some concerns
**Sekiya 2001**	**Some concerns**	**Some concerns**	**Low**	**Low**	**Some concerns**	**Some concerns**
**Tolman 2009**	**Some concerns**	**Low**	**High**	**Low**	**Low**	**High**
**Fofonka 2018**	**Some concerns**	**Some concerns**	**Low**	**Low**	**Some concerns**	**Some concerns**
**Sposito 2021**	**Low**	**Some concerns**	**Low**	**Low**	**Low**	**Some concerns**
**Koren 2012**	**Some concerns**	**Some concerns**	**Some concerns**	**Low**	**Some concerns**	**Some concerns**

Note: D1, bias arising from the randomization process; D2, bias due to deviations from intended interventions; D3, bias due to missing outcome data; D4, bias in measurement of the outcome; D5, bias in selection of the reported result. **The five narrative-only reports newly assessed in response to peer review are shown in bold. Wolf et al. (2021) and Sposito et al. (2021) are separate reports from the same ADDENDA-BHS2 trial. Overall, 11 of 19 report-level results (57.9%) were at high risk of bias, and eight (42.1%) raised some concerns; none were at overall low risk.**
